# Sapling recruitment as an indicator of carbon resiliency in forests of the northern USA

**DOI:** 10.1002/ece3.70077

**Published:** 2024-08-07

**Authors:** Lucas B. Harris, Christopher W. Woodall, Anthony W. D'Amato

**Affiliations:** ^1^ Rubenstein School of Environment and Natural Resources University of Vermont Burlington Vermont USA; ^2^ USDA Forest Service, Research and Development Inventory Monitoring and Assessment Research Durham New Hampshire USA

**Keywords:** carbon stocks, forest resilience, northern temperate and boreal forest, permanent plot, sapling recruitment, tree regeneration

## Abstract

Tree regeneration shapes forest carbon dynamics by determining long‐term forest composition and structure, which suggests that threats to natural regeneration may diminish the capacity of forests to replace live tree carbon transferred to the atmosphere or other pools through tree mortality. Yet, the potential implications of tree regeneration patterns for future carbon dynamics have been sparsely studied. We used forest inventory plots to investigate whether the composition of existing tree regeneration is consistent with aboveground carbon stock loss, replacement, or gain for forests across the northeastern and midwestern USA, leveraging a recently developed method to predict the likelihood of sapling recruitment from seedling abundance tallied within six seedling height classes. A comparison of carbon stock predictions from tree and seedling composition suggested that 29% of plots were poised to lose carbon based on seedling composition, 55% were poised for replacement of carbon stocks (<5 Mg ha^−1^ difference) and 16% were poised to gain carbon. Forests predicted to lose carbon tended to be on steeper slopes, at lower latitudes, and in rolling upland environments. Although plots predicted to gain and lose carbon had similar stand ages, carbon loss plots had greater current carbon stocks. *Synthesis and applications.* Our results demonstrate the utility of considering tree regeneration through the lens of carbon replacement to develop effective management strategies to secure long‐term carbon storage and resilience in the context of global change. Forests poised to lose C due to climate change and other stressors could be prioritized for regeneration strategies that enhance long‐term carbon resilience and stewardship.

## Graphical abstract

We examined the implications of tree regeneration patterns for future aboveground forest carbon dynamics across forests of the eastern and midwestern USA. We found substantial variability in the potential for tree regeneration to replace existing carbon stocks, as well as associations between carbon replacement and physiographic, geographic, and stand‐level characteristics. Our approach of using tree regeneration patterns as an indicator of potential carbon dynamics has implications for managing tree regeneration to secure long‐term forest carbon storage.

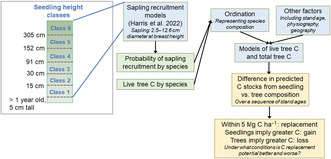

## 1 INTRODUCTION

Tree regeneration is a key component of forest resilience, which is commonly defined as the degree of perturbation a system can withstand and still return to its previous state as opposed to shifting to a new state (Holling, 1973; Millar et al., 2007). Successful tree regeneration and recruitment in temperate forests are threatened by widespread and varied challenges include climate change, altered disturbance regimes, over‐browsing, pests, and pathogens (Dey et al., 2019). These regeneration challenges can drive shifts in forest composition over time, or even lead to regeneration failure following disturbance (Cerioni et al., 2024; Davis et al., 2023; Miller & McGill, 2019; Moser et al., 2010). Both despite and because of the high degree of uncertainty involved in predicting forest trajectories from tree regeneration patterns, a pressing need exists to develop improved tree regeneration indicators to inform forest management and policy (Harris et al., 2022; Vickers, McWilliams, Knapp, D'Amato, Saunders, et al., 2019). Tree regeneration is a major source of uncertainty in predicting forest trajectories including carbon (C) dynamics because regeneration tends to be represented coarsely in models of forest dynamics (Anderegg et al., 2020; Díaz‐Yáñez et al., 2024; Millington et al., 2013).

As forests are a globally important carbon (C) sink (Harris et al., 2021) their role as a natural climate solution is a growing focus of forest management and larger discussions surrounding climate mitigation policies (Daigneault et al., 2022; Domke et al., 2020). Long‐term forest C stocks and sequestration rates depend strongly on both resistance to mortality events as well as resilience. Resistance has been widely discussed in the context of C stability, particularly the need to pursue management and policy strategies focused on prioritizing forest resistance to mortality under likely future conditions rather than seeking to maximize short‐term C stocks (D'Amato et al., 2022; Hurteau et al., 2019). Yet the implications of forest resilience on long‐term C dynamics, especially the capacity of tree regeneration to eventually replace C stocks reduced through tree mortality, have received comparatively less attention (Anderegg et al., 2020). The potential consequences of tree regeneration patterns are particularly important given non‐native pests and disturbances, such as wildfire and drought, have already increased tree mortality rates and threaten to reduce the strength of the forest C sink (Fei et al., 2019; McDowell et al., 2020). Interest in managing tree regeneration has also surged, especially planting trees for climate mitigation (Clark et al., 2023; Holl & Brancalion, 2020).

**FIGURE 1:**
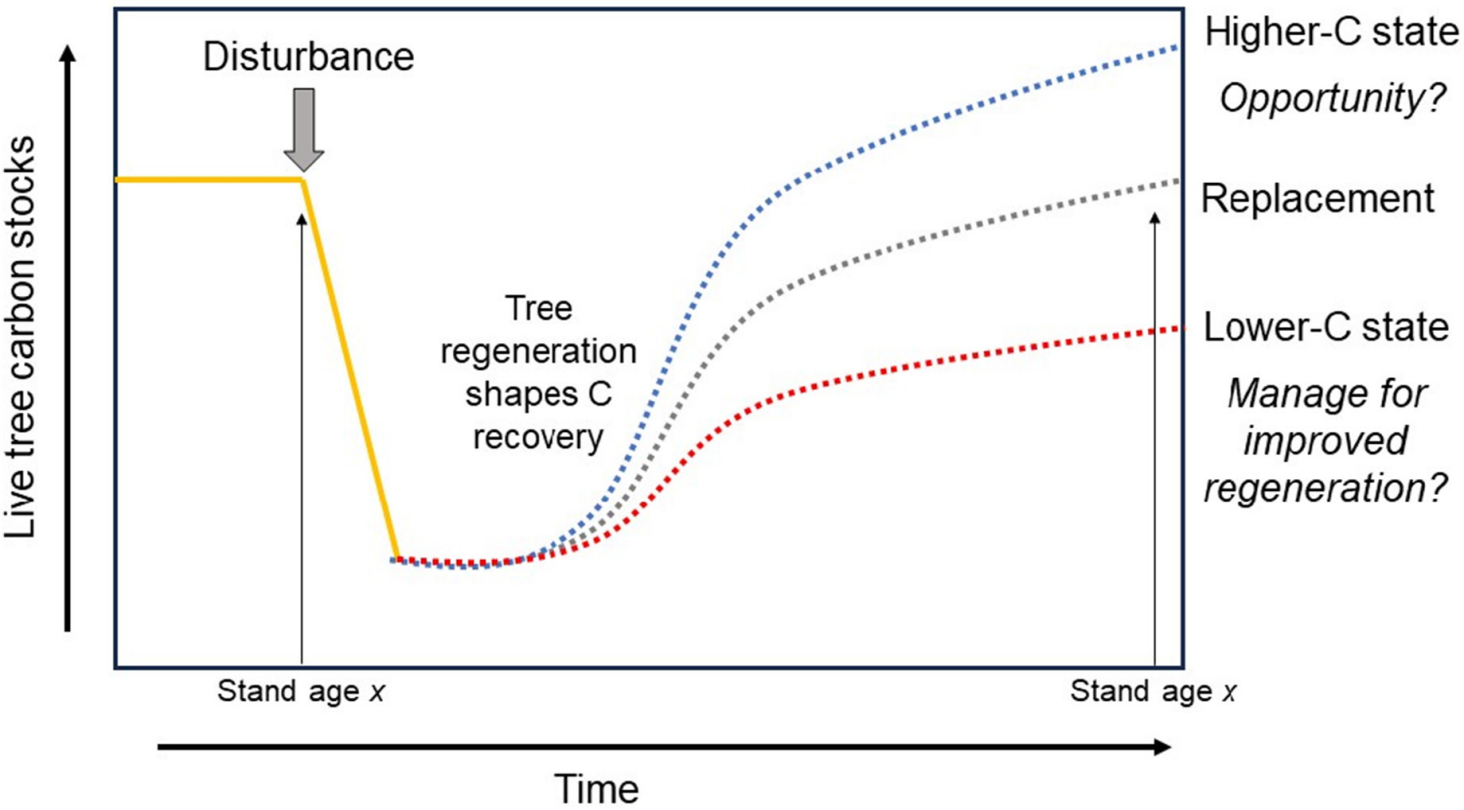
Conceptual diagram illustrating aboveground forest carbon (C) replacement and its connection to tree regeneration. Live tree C is shown to simplify the diagram, as disturbance will shift C stocks from live to dead C pools with variable rates of C emissions over time.

Evaluating the species composition of trees compared to that of seedlings or saplings through a lens of “C replacement” (Figure 1) may be a useful framework to assess whether current tree regeneration patterns are consistent with potentially high rates of C sequestration in the future and what management actions (i.e., carbon stewardship) might encourage resilient future C stocks. However, two major challenges exist in defining and analyzing C replacement potential. First, empirical analyses of how tree species composition affects C storage are surprisingly rare with analyses comparing seedling to tree composition from a C perspective being even rarer (e.g., Knott et al., 2023). Using seedling composition to develop indicators of possible future C dynamics may provide valuable information for managing regeneration while potentially informing stand‐based growth‐and‐yield models such as the Forest Vegetation Simulator (FVS) (Crookston & Dixon, 2005) that are commonly used to predict future C stocks and sequestration (Anderegg et al., 2020; Herbert et al., 2023). Second, linking seedling species composition to future tree composition is challenging due to highly variable seedling mortality rates. The uncertainty associated with this second issue can be reduced by quantitatively linking seedling tallies with survival and recruitment of saplings and trees (Harris et al., 2022, 2024).

Here, we apply the concept of C replacement (Figure 1) by using existing tree regeneration patterns to develop an indicator of potential changes to future C stocks across forests of the northeastern and midwestern USA using field plots from the national forest inventory. To better link seedling abundance to future tree composition and associated C attributes, we used a subset of USA Forest Service Forest Inventory and Analysis (FIA) plots that are systematically sampled across six height classes (McWilliams et al., 2015) and applied a recently developed method that assesses the likelihood of near‐term sapling recruitment based on seedling abundance (Harris et al., 2022). Here we define seedlings using an upper size limit of 2.5 cm Diameter at Breast Height (DBH; 1.3 m height) following FIA conventions (Bechtold & Patterson, 2005), although we acknowledge that the definition of a seedling varies widely and that the FIA threshold is large compared to commonly used thresholds of <50 cm or < 100 cm tall (Martini, 2024). Our goal was to identify where and under what conditions (forest type, physiographic, and geographic setting) potential sapling recruitment, as inferred through quantification of tree regeneration, suggests increased or decreased future aboveground C stocks. Although we expected considerable heterogeneity in C replacement potential, we had three main expectations:

1. A combination of heavy browsing, invasive species, and changing disturbance regimes are known to threaten regeneration of key late‐successional species that are long‐lived and attain large size like sugar maple (*Acer saccharum* Marsh.) and oak (*Quercus*) species (Bose et al., 2017; Miller & McGill, 2019). Therefore, we expect that many late‐successional stands dominated by these species should be poised to lose C based on regeneration and potential sapling recruitment patterns.
2. Compositional mismatch between trees and regeneration may result from successional processes. We expect that replacement of early successional species with late‐successional species that are long‐lived and capable of attaining greater size will tend to lead toward greater C stocks over time. Therefore, stands dominated by early to mid‐successional species should be poised for C gain based on regeneration and may represent opportunities to augment future C stocks (Knott et al., 2023).
3. In forest types that historically were maintained by disturbance such as fire (e.g., pine and oak/pine forests) we expect regeneration attributes to suggest C gain due to ongoing mesophication leading to denser stands of shade‐tolerant species (Nowacki & Abrams, 2008; Zhao et al., 2021). However, gains in C stocks due to mesophication must be weighed against loss of ecological function and cultural value in these stands (Littlefield & D'Amato, 2022).

## 2 METHODS

**FIGURE 2:**
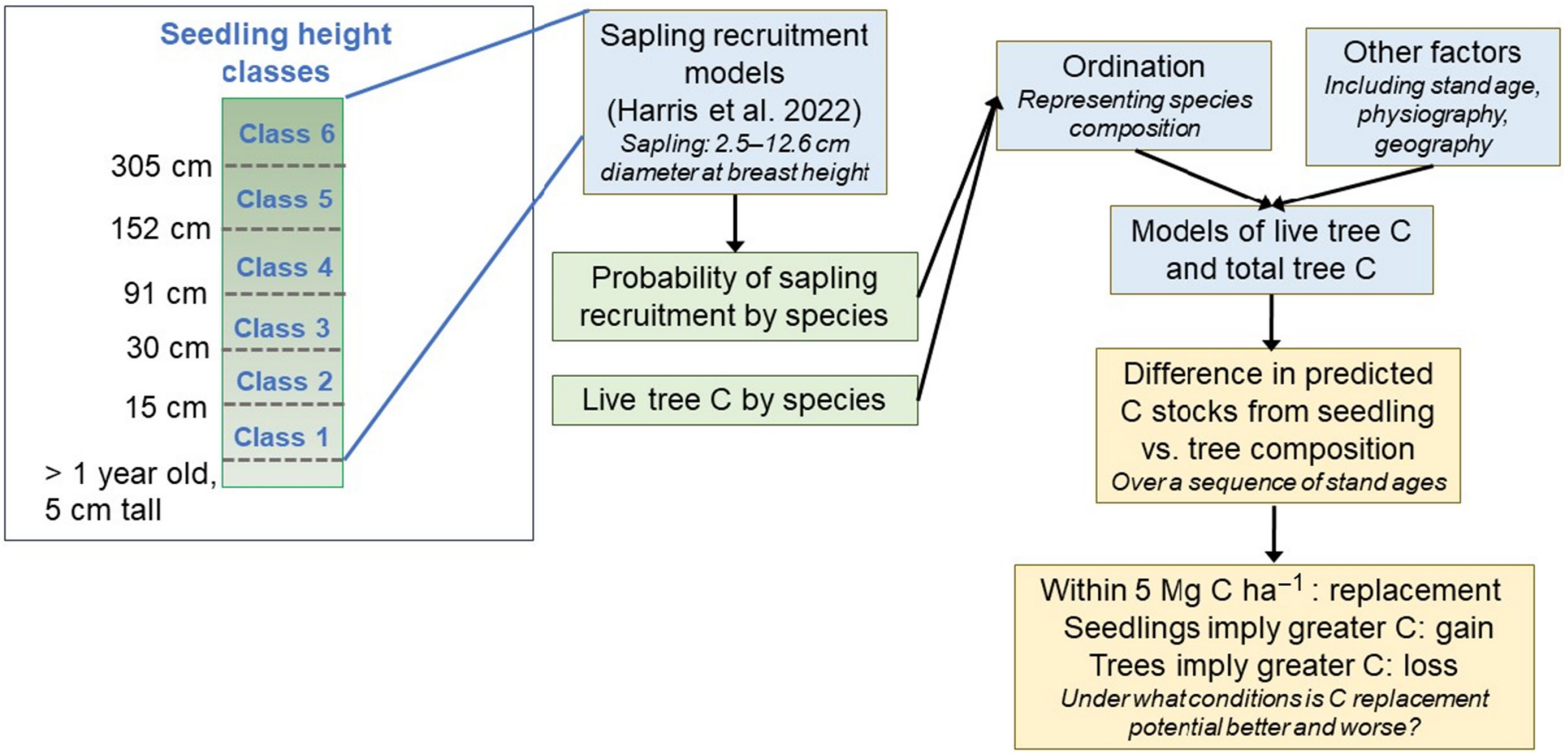
Outline of methodology used to relate tree species composition to carbon (C) stocks and assess the implications of tree regeneration and associated sapling recruitment patterns for C replacement using forest inventory plots in which seedling abundance is tallied within six different height classes.

To analyze aboveground C storage and tree regeneration we used FIA's Regeneration Indicator (RI) dataset. Our approach is summarized in Figure 2. The Northern Research Station began RI data collection in 2012 for a subset of 1/8 of all FIA field plots across the USA Midwest and Northeast, or an intensity of one plot per ~194 km^2^. We used the RI dataset as opposed to the broader pool of FIA plots primarily due to its more detailed seedling surveys that were designed to provide more robust assessments of tree regeneration (McWilliams et al., 2015). Per RI protocols, tree seedlings are tallied by six different height classes within each microplot instead of FIA's standard single size class (McWilliams et al., 2015). RI measurements also coincide with a detailed vegetation profile and litter and duff measurements that can be associated with regeneration patterns (McWilliams et al., 2015). Another advantage to using the RI dataset is that downed dead wood (Downed Woody Material, DWM) is also inventoried for this same subset of plots, enabling measurement‐based C estimates to be calculated for DWM as opposed to the modeled values available for most FIA plots (Woodall et al., 2019). FIA plots in this region are remeasured at ~5‐year intervals. Here, we refer to the most recent measurement as “Time 2” and the prior measurement as “Time 1”.

**FIGURE 3:**
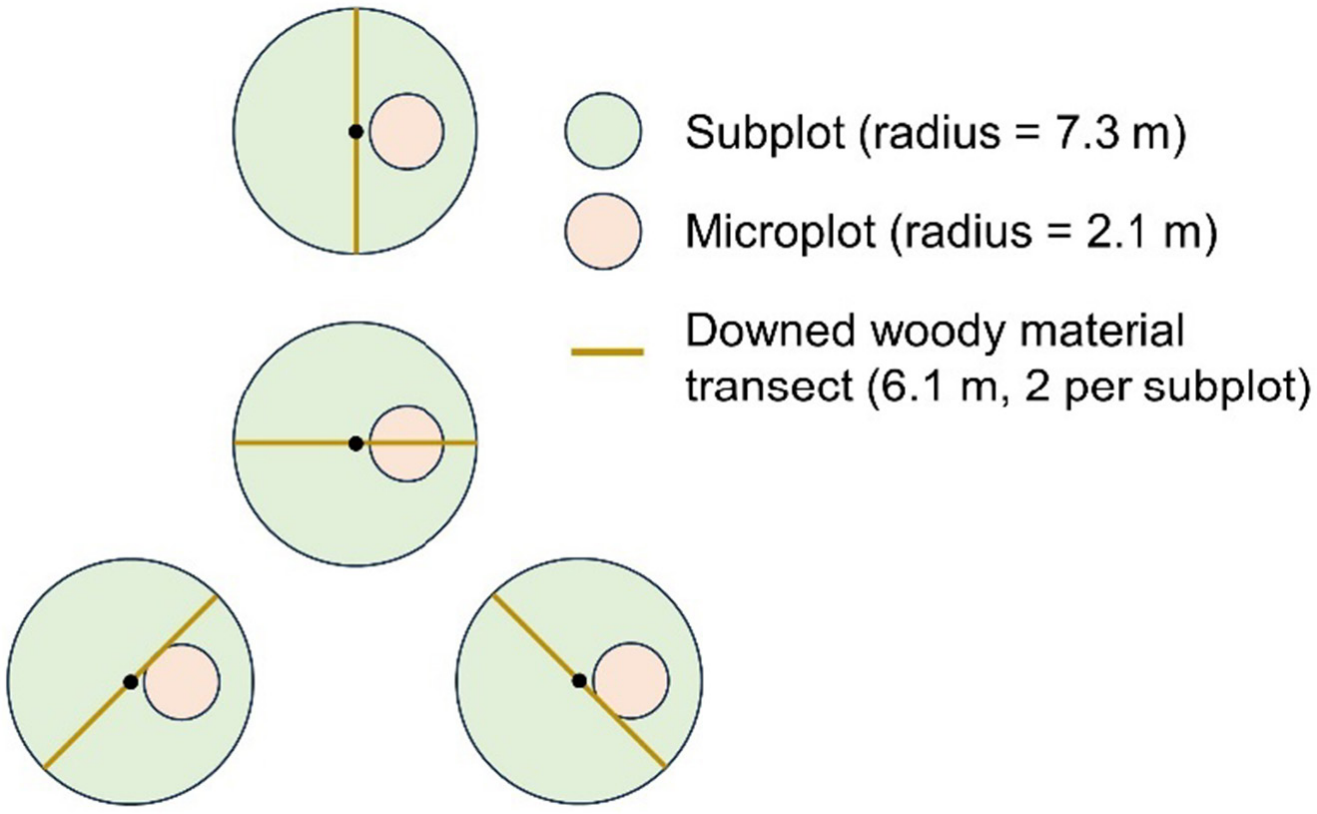
Illustration of the Forest Inventory and Analysis plot design used in this analysis.

Within each plot, one 168 m^2^ subplot is located at the plot center and three more subplots are surveyed 36.6 m away from plot center at 0°, 120°, and 240° (Figure 3). Each subplot contains a circular microplot of 13.5 m^2^ located 3.7 m plot the subplot center at a 90° azimuth. Trees ≥12.7 cm DBH are surveyed in the subplots whereas seedlings (<2.5 cm DBH) and saplings (2.5–12.6 cm DBH) are surveyed in the microplots (Bechtold & Patterson, 2005). Site attributes such as forest type or stand age are spatially mapped within each plot and can be considered a study observation within each plot henceforth referred to as plot conditions.

FIA plots were selected from the FIA database (Gray et al., 2012) for analysis based on the following criteria: (1) All four subplots had RI seedling data from at least two time periods (*n* = 1563 plots); (2) the entirety of the plot lay on accessible forest land as defined by FIA (Bechtold & Patterson, 2005); (3) artificial regeneration was not present; (4) the plot had been completely sampled for DDW; and (5) no trees were identified as cut and removed in the most recent measurement, to focus on natural dynamics in non‐harvested plots (*n* = 1162 plots after all these criteria were applied).

### 2.1 Carbon calculations

C in live trees and standing dead trees was obtained from FIA's stem‐level estimates of aboveground C, which are calculated by the FIA program using the National Scale Volume and Biomass Estimators (NSVB) system released in October 2023 (Westfall et al., 2024). In this system, allometric models of different forms are used to estimate aboveground biomass from tree DBH and height. These models are developed by species and ecoregion (*n* = 6 regions overlapping the study area) based on a combined database of measurements from >200,000 sampled trees. C is calculated from biomass using species‐specific estimates of C fractions from either direct measurements or estimates based on specific gravity (Westfall et al., 2024). Belowground C was not considered in this analysis because it is not measured under standard FIA field protocols or RI protocols. Under current FIA protocols (post‐2011) DWM is measured along two transects per subplot (eight per plot, Figure 3) including coarse woody material (CWM, ≥ 7.6 cm diameter) along the entirety of each 6.09‐m transect and fine woody material <2.5 cm and 2.5–7.6 cm diameter along 1.83 and 3.05 m of each transect, respectively (Woodall et al., 2019). The FIA program then calculates biomass and C from DWM components using a set of species‐level and forest type group‐level parameters (Woodall et al., 2019). C in DWM was calculated using condition‐level calculations that were scaled to the plot level using the proportion of the plot in each condition. For analysis, live tree C was calculated both by species and across species for each plot, and “total C” (i.e., C in live trees, standing dead trees, and DWM), was calculated at the plot level. Change in live tree C was converted to rate of change by dividing by the remeasurement period noted in the FIA database (4.7–8.1 years, mean of 6.0 years).

### 2.2 Species composition

Proportional species composition in each RI plot was calculated in two ways: C stocks (“tree C composition”) and likelihood of sapling recruitment based on seedling abundance (“recruitment composition”). We used this sapling recruitment likelihood instead of relative seedling abundance because the former provides a more robust assessment of potential outcomes of observed seedling abundance and therefore should lead to more accurate assessments of C replacement potential (Harris et al., 2022). Tree C composition was the proportion of live tree aboveground C that each species comprised. Likelihood of sapling recruitment was calculated following Harris et al. (2022), which is a method that links seedling abundance by RI height class to later sapling recruitment (Harris et al., 2022). The method involves developing Boosted Regression Tree (BRT) models (Elith et al., 2008) to predict the presence of newly recruited saplings in a given subplot measurement as a function of seedling abundance from RI height classes in the previous measurement and other variables representing terrain, climate, vegetation, and substrate. Models were developed for 15 individual species with a sufficient sample size (>30 subplots in which sapling recruitment was present, Table S1). For other species (*n* = 139), a general model was developed. Evaluations of model accuracy supported the findings of Harris et al. (2022) that the use of RI seedling height improves predictions of sapling recruitment as compared with standard FIA seedling tallies in one size class (Table S2). The general model of all species combined was only marginally less accurate than most of the individual species models (Table S2), indicating that robust estimates of recruitment likelihood could be produced from a general model as well as the individual species models. Recruitment model development and evaluation are detailed in the Appendix S1.

Recruitment models were used to generate the probability of sapling recruitment for each species in each subplot based on Time 2 (most recent) RI seedling abundance. If no seedlings of a given species were present, the probability of recruitment for that species was assumed to be zero. Recruitment probabilities were averaged at the plot level and relativized among species to generate recruitment composition scores that represent relative recruitment probabilities. For example, if a plot had sapling recruitment probabilities of 0.8 for *Acer saccharum*, 0.7 for *Fagus grandifolia*, 0.5 for *Betula alleghaniensis*, and no seedlings of any other species, recruitment composition values for these species would be 0.4, 0.35 and 0.25 respectively. The result was a novel metric of potential sapling recruitment composition that provides a more accurate assessment of near‐term stand trajectories than raw seedling abundance.

To enable species composition of trees and seedling to be compared with one another, Nonmetric Multidimensional Scaling (NMDS) ordination was used to quantify and visualize species composition based on live tree C and recruitment composition at Time 2. Two steps were taken to help construct a robust and interpretable ordination. First, species which represented <1% of both overall live tree C and recruitment composition (*n* = 118 species) were collapsed into an “other species” category (Table S3). Second, we used FIA forest type group classifications (Burrill et al., 2021) to remove groups with <20 plots, leaving seven common forest type groups (Table 1). Because forest types and type groups are classified at the condition level (Burrill et al., 2021), we assigned such classifications to individual plots based on the condition within the plot that represented the largest proportion of the plot and used the condition at the plot center in the case of ties. In addition, two forest types within the white/red/jack pine group were removed (red pine and jack pine) because we found that plots representing these forest types produced outliers in the ordinations. Best practices for constructing ordinations are widely debated (Poos & Jackson, 2012), including treatment of rare species/groups and outliers. Jack pine (*Pinus banksiana*) and red pine (*Pinus resinosa*) are both fire‐associated species that reach their southern range limit within our study area and stands dominated by these species may have been too compositionally distinct to be well‐represented in the ordination given their ecology and small sample size (*n* = 10 and 14 respectively) within the plots used in this study. Removing these plots left 1076 of the original 1162 RI plots that met our criteria, including 1075 plots with nonzero live tree C and 1068 plots with at least one seedling present (therefore *n* = 2143 entries in the ordination). These plots contained an average of 6.5 species (standard deviation [SD] = 2.5) in the live tree layer and 6.9 (SD = 3.5) species per plot for seedlings across the six height classes in the most recent (Time 2) measurement, including the uncommon species combined into the “other species” category. In the previous measurement (Time 1) these plots averaged 6.4 (SD = 2.6) species per plot for live trees and 7.4 (SD = 3.7) species per plot for seedlings. NMDS ordinations were run using the “metaMDS” function in the “vegan” R package (Oksanen, 2022). The Bray–Curtis dissimilarity index was used, the maximum number of iterations within a run was set to 999 and the best solution was chosen from 250 random starts. Four dimensions were used to maintain interpretability while reducing stress (0.13 in the final NMDS ordination) to an acceptable level (i.e., <0.2, Kruskal, 1964).

**TABLE 1:** Summary of forest inventory plots used in the analysis by forest type group, including mean (standard deviation) of carbon (C) stocks.

Forest type group	Plots	Live tree C (Mg C ha^−1^)	Total tree C (Mg C ha^−1^)^a^
Aspen/birch	113	48.2 (24.8)	56.2 (26.5)
Elm/ash/cottonwood	74	61.6 (34.9)	71.9 (39.3)
Maple/beech/birch	304	73.2 (36.1)	85 (44.9)
Oak/hickory	317	75.3 (36.8)	84.7 (38.9)
Oak/pine	34	72.4 (32.4)	81.3 (34.7)
White/red/jack pine	36	88.6 (36)	100.1 (38.1)
Spruce/fir	198	45.7 (25.5)	55.5 (31.2)
All groups	1076	65.8 (35.7)	76.0 (40.5)

^a^ Aboveground carbon in live trees, standing dead trees and downed woody material.

### 2.3 Models of carbon stocks

After performing the ordination to represent species composition, Random Forest models (Breiman, 2001) were developed to link tree species composition to C stocks, enabling assessment of C replacement potential by comparing C stock predictions based on tree as opposed to recruitment composition. These models isolated the effect of species composition from other potential determinants of C stocks such as stand age. Two response variables were considered: (1) live aboveground tree C stocks in Time 2 (“live C”) and (2) total aboveground tree C stocks in live trees, standing dead trees, and DWM (“total C”). Predictors included ordination scores for tree C composition from each of the four ordination axes, stand age, terrain, climate and recent disturbance and treatment history (Table 2). Each model was built using 1000 regression trees and otherwise default settings in the “randomForest” R package (Liaw & Wiener, 2002). Variable importance was quantified as the increase in mean standard error (normalized by standard deviation) that occurred when individual variables were randomly permuted (Breiman, 2001). Partial dependence plots, implemented in the “pdp” R package (Greenwell, 2017) were used to visualize the effects of predictor variables on C storage. We compared models that were run using either three climate variables (1991–2020 mean annual temperature, total precipitation and vapor pressure deficit) derived from gridded climate data (Daly et al., 2008, 2015) or latitude and longitude. We selected the models using latitude and longitude as they had greater accuracy based on pseudo‐*r* ^*2*^ calculated from out‐of‐bag data. We also attempted to build models of C stock change (change in live tree C and total C from Time 1 to Time 2) but obtained very low model accuracy (pseudo‐*r* ^*2*^ < 10%).

**TABLE 2:** Variables used in models of carbon (C) stocks.

Variable	Source^a^	Details
Tree species composition	Ordination scores	C composition values from each of 4 axes
Stand age	Condition	
Physiographic class	Condition	
Forest type group	Condition	
Elevation	Plot	
Slope	Condition	
Aspect	Condition	
Land ownership	Condition	Private, USDA Forest Service, other federal land, or state/local
Disturbance type	Condition	None, insects, disease, fire, animals, weather, vegetation, human and unknown
Latitude	Plot	
Longitude	Plot	

^a^ Name of table from Forest Inventory and Analysis database, or species compositional scores from the ordination.

C stock models were used to quantify the potential for C replacement at the plot level by comparing predictions of C stocks using tree composition vs. recruitment composition at equivalent stand ages. Because the live C model had higher accuracy than the total C model but the two models were otherwise similar, predictions were generated from the live C model for this analysis. Predicted C stock values were generated using ordination axis scores from (1) live tree C composition (which was used to build the models) and (2) recruitment composition (based on sapling recruitment likelihood). We generated these predictions for each plot with stand age set to 100 years and other variables (apart from ordination scores) kept at their observed values. A sensitivity analysis demonstrated that results obtained at a stand age of 100 years also held across a range of maturing to mature stand ages of 60–140 years (see Appendix S1). The difference between predicted C stocks using tree C scores and sapling recruitment likelihood scores was used to quantify C replacement potential. To simplify the results, we divided plots into three categories: “replacement” plots had ≤5 Mg ha^−1^ difference in predicted C stocks, whereas “loss” and “gain” plots were those in which recruitment composition implied >5 Mg ha^−1^ loss or gain in C stocks, respectively. Results were robust to the use of categorized vs. non‐categorized difference in predicted C stocks (see Appendix S1).

To identify factors associated with C replacement potential and to evaluate the extent to which our expectations for C replacement potential were met, the site and stand‐level variables which differed significantly by C replacement category (loss, replacement and gain) were assessed using Kruskal‐Wallis tests for continuous variables and Chi‐square tests for factors. A Holm‐Bonferroni correction was applied for multiple comparisons. The following variables were considered: latitude, longitude, elevation, slope, aspect, live tree C stocks, standing dead tree C stocks, CWM C stocks, change in live tree C from Time 1 to Time 2, mean annual temperature, annual precipitation, vapor pressure deficit, ownership group, physiographic class, disturbance type, and forest type group. Results are shown only for variables with significant differences (*p* < .05).

## 3 RESULTS

### 3.1 Carbon stocks and species composition

**FIGURE 4:**
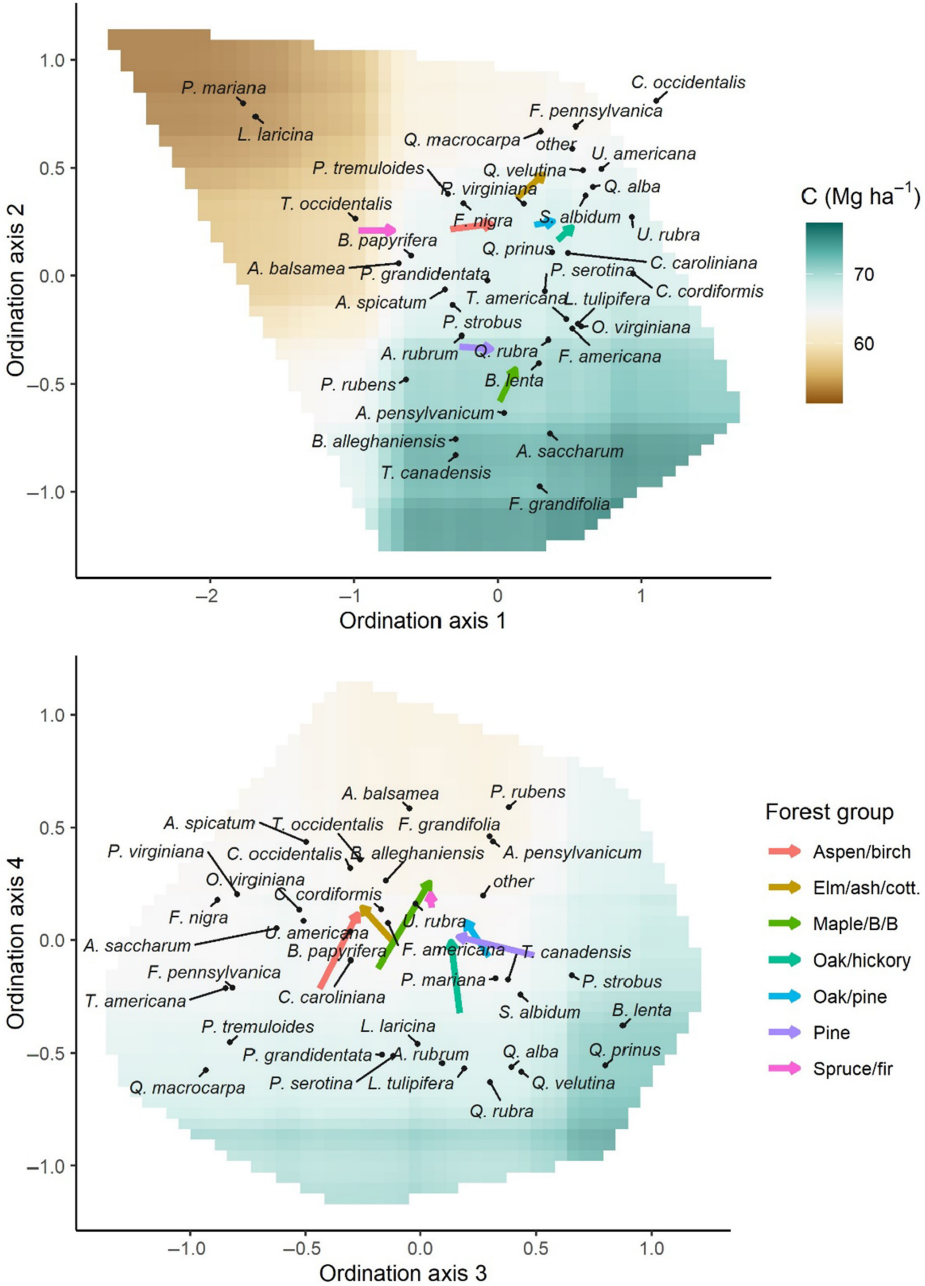
Ordination scores for tree species, and partial dependence plots displaying the marginal influence of species composition on predicted live aboveground tree carbon (C) stocks. Arrows show average species composition of live tree C (beginning of arrow) and recruitment composition (end of arrow) by forest type group. Longer arrows represent forest type groups where the composition of live tree C varied more from the composition of seedlings.

Live tree C stocks averaged 65.8 Mg ha^−1^ and total C (including snags and DWM) averaged 76.0 Mg ha^−1^ with high variability among forest type groups (Table 1). The model of live tree C stocks had a pseudo‐*r* ^*2*^ of 41.8%, as compared with 36.9% for a model of total C. Stand age had the strongest influence on live C stocks, followed by latitude (Figure S1). Ordination axes 1 and 2 were the third and sixth most important variables, respectively, with physiographic setting fourth and longitude fifth. The other 10 variables, including ordination axes 3 and 4, were of low–moderate importance. Variable importance in the total C model was very similar to that of the live C model (Figure S2). The two models were also highly similar in terms of the shape of relationships with each predictor, as shown by partial dependence plots (Figures S3 and S4). Bivariate partial dependence plots of the ordination axis values (Figure 4) suggested that live C stocks tended to be greater in plots dominated by *Acer saccharum*, *Tsuga canadensis*, and *Fagus grandifolia*, and lowest in stands with *Picea mariana* and *Larix laricina*. Ordination axes 3 and 4 indicated that C stocks tended to be greater in oak‐dominated (*Quercus* sp.) plots (Figure 4).

### 3.2 Recruitment composition and carbon replacement

**FIGURE 5:**
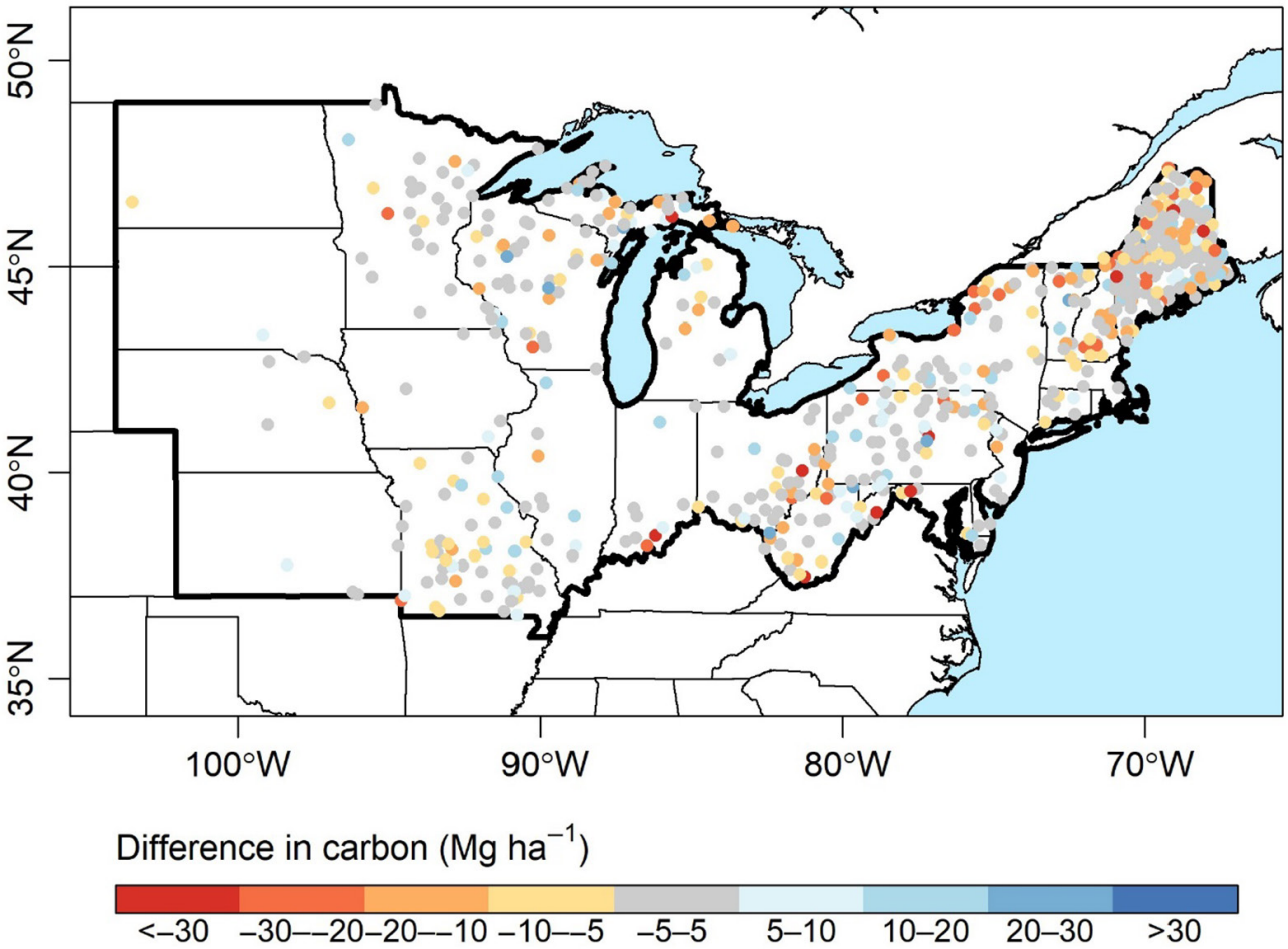
Difference in predicted aboveground live tree carbon stocks when using recruitment composition (predicted likelihood of sapling recruitment) as opposed to current tree species composition, at a stand age of 100 years. Blue (red) values indicate greater (lesser) predicted carbon storage based on recruitment composition.

**FIGURE 6:**
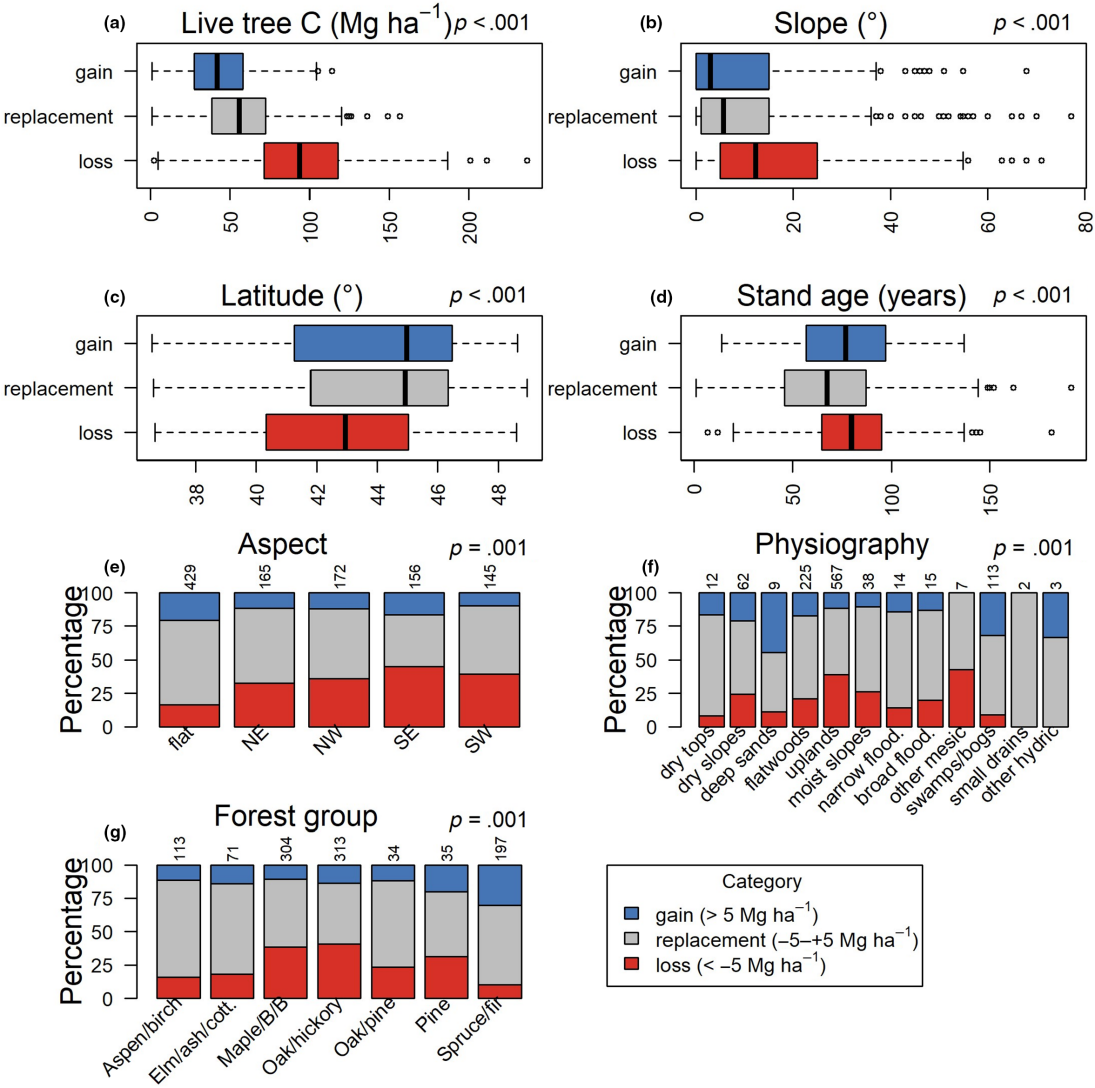
Variation in carbon (C) replacement, i.e., plots predicted to lose, replace or gain C stocks based on recruitment composition compared to tree composition at a predicted stand age of 100 years, by physiography, geography, and stand attributes: (a) live tree C, (b) slope, (c) latitude, (d) stand age, (e) aspect, (f) physiographic setting and (g) forest type group. Variables are ordered alphabetically by significance (Kruskal–Wallis test for numeric variables and Chi‐squared test for categorical variables) and *p*‐values (Holm‐Bonferroni correction applied) are reported in each panel. For categorical variables, number of plots in each category is shown above each bar. Flood. = floodplain, Elm/ash/cott. = Elm/ash/cottonwood, and Maple/B/B = Maple/Beech/Birch.

Overall, 55% of plots were within the category of C replacement whereas 29% and 16% were within the C loss and gain categories, respectively. Although the mean difference in predicted C stocks between recruitment and tree composition was small (−2.2 Mg ha^−1^), we observed considerable variability (SD = 7.7 Mg ha^−1^) and spatial heterogeneity (Figure 5). Plots that were predicted to lose C based on recruitment composition tended to be farther south and be located in hillslope and rolling upland environments (Figure 6b–c, e–f). C loss plots tended to have lower current C stocks than C gain plots (Figure 6a), yet C gain and loss plots were similar in terms of stand age whereas C replacement stands tended to be younger (Figure 6d). Whether plots were predicted to gain or lose C storage based on recruitment composition also varied by forest type group, with the lowest C replacement and gain prospects in maple/beech/birch and oak/hickory forests and the greatest prospects in spruce/fir forests (Figure 6g).

## 4 DISCUSSION

Tree regeneration and associated sapling recruitment is increasingly a key consideration of forest management, including adjusting silvicultural systems to improve natural regeneration (Kenefic et al., 2021; Rogers et al., 2022) and planting trees for climate adaptation and mitigation (Clark et al., 2023). Given limited resources, management for improved tree regeneration must be targeted where interventions will be the most effective at increasing forest resilience and adaptive capacity (Webster et al., 2018). The indicator and broader concept of C replacement that we explore in this work is useful for identifying stand and site‐level conditions under which managing for regeneration might confer the greatest benefit in terms of securing resilient forest C stocks. Although the mean difference between projected C stocks based on seedling as opposed to tree composition was small, we found substantial variability in C replacement potential by physiographic setting, forest type group and current C storage that together suggest both challenges and opportunities. Forests poised to lose C due to transfers to the atmosphere or other pools via tree mortality without concomitant sapling recruitment could be prioritized when developing landscape to regional‐scale management strategies, and critically assessed to determine how regeneration could be managed to increase C resilience. Although we did not attempt to develop robust predictions of future C stocks, our results underscore the need to better understand and predict tree regeneration patterns from a C dynamics perspective (Anderegg et al., 2020) that could inform future, refined modeling exercises.

Our results are consistent with our first expectation, that stands dominated by late‐successional species with known regeneration challenges such as sugar maple and oaks (Bose et al., 2017; Miller & McGill, 2019) would have low C replacement potential. Oak/hickory forests had the greatest potential for C loss in our analysis with results indicating shifts away from oak species toward other hardwoods. The circumstance that oaks are underrepresented in the regeneration layer relative to the overstory in the eastern USA, along with shifts toward mesophytic species in mixed‐oak stands, has long been an issue of concern and is due to a complex assortment of factors including insufficiently frequent or intense disturbance (Brose et al., 2013; Larsen & Johnson, 1998; Woodall et al., 2008). Maple/beech/birch forests had the second‐highest potential for C loss, and we found that seedling composition was shifted away from sugar maple and toward other species including American beech (*Fagus grandifolia* Ehrh.). In maple/beech/birch forests, sugar maple (associated with high C stocks in our analysis) is often abundant among small seedling size classes yet fails to meet stocking criteria for larger seedlings and saplings due to factors including heavy browsing by white‐tailed deer (*Odocoileus virginianus*) and interference from shrubs and understory trees (Henry et al., 2021). It is also notable that the maple/beech/birch and oak/hickory groups have the greatest degree of field‐estimated browsing impacts among forest type groups in the study region (McWilliams et al., 2018). Overall, our results are consistent with the idea that regeneration challenges for sugar maple and oak species will negatively affect future forest C storage.

Evidence was mixed for our second main expectation, that ongoing successional processes might lead to C gain potential in forests where overstories are dominated by early to mid‐successional species (Knott et al., 2023). Consistent with this expectation, the two forest groups dominated by early to mid‐successional species (aspen/birch, elm/ash/cottonwood) also had stronger C replacement prospects than the forest groups associated with late‐successional, longer‐lived species (maple/beech/birch, oak/hickory). Also, we found that stands with currently low aboveground C stocks were poised to gain C on average based on seedling composition. Counter to this expectation, however, C loss and gain plots had similar stand age distributions. Therefore, our results suggest, perhaps counterintuitively, that challenges with C replacement are most acute in more productive settings (as indicated by high C stocks in relation to stand age). Regeneration may be suppressed in productive sites that achieve canopy closure, but because our methodology relies on relative composition of regeneration rather than absolute abundance, our results indicate challenges with C replacement in productive stands based on seedling species composition rather than low abundance.

Finally, we expected forest types historically associated with disturbance to be poised for C gain on average due to ongoing forest mesophication in the absence of disturbance. In support of this idea, we found that oaks were underrepresented in the seedling layer of oak/hickory forests resulting in poor C replacement potential. Also, fire‐prone physiographic settings (dry slopes and ridges and deep sands) had better C replacement prospects than most settings. However, pine and oak/pine forests were more likely to lose than gain C based on seedling composition. Eastern white pine (*Pinus strobus* L.) accounted for the majority of pine and oak/pine plots in our analysis and is a long‐lived tree capable of attaining large size and high stand‐level carbon stocking (Abrams, 2001; Waterman et al., 2020). Therefore, compositional shifts away from white pine may lead to lower C stocks. To the extent that disturbance‐associated forest types are poised for C gain due to mesophication, the potential to increase C stocks must be considered in the context of tradeoffs with other ecosystem benefits (Littlefield & D'Amato, 2022; Zhao et al., 2021).

Although standing dead trees and DWM comprised a substantial 10 Mg C ha^−1^ on average in our analysis, we found that modeling total aboveground tree C (live tree C and deadwood C) resulted in a less accurate but otherwise highly similar model compared with a model of live C only. In agreement with prior work indicating that C in deadwood is highly variable and difficult to predict (Woodall et al., 2013, 2015), these results suggest that uncovering empirical associations with deadwood C is a difficult task and furthermore that considering tree species composition may not help to predict deadwood C storage. Additionally, although we did not consider belowground C, it could potentially be considered in future work as inventory‐based estimates of soil organic C continue to be improved (Domke et al., 2017).

We conducted our analysis under a working hypothesis that composition of tree regeneration tied to sapling recruitment is an indicator of near‐term species dynamics. This hypothesis underpins a body of literature using differences between tree and seedling presence or abundance to indicate species range shifts (Bell et al., 2014; Lenoir et al., 2009; Woodall et al., 2009) and potential changes in composition (Knott et al., 2023; Miller & McGill, 2019). Site‐specific studies within our study region do suggest that tree seedling patterns, and advance regeneration in particular, often dictate near‐term forest dynamics following disturbance (Leak, 2007; Plotkin et al., 2013). By adjusting seedling abundance to account for the influence of seedling height class and other stand and site‐level factors on expected sapling recruitment, we went a step further than studies comparing seedling to tree abundance. Greater survival and sapling recruitment rates for large‐sized seedlings as compared with small seedlings make accounting for seedling size important when assessing how regeneration may affect future forest dynamics (Harris et al., 2022; McWilliams et al., 1995, 2015; Vickers, McWilliams, Knapp, D'Amato, Dey, et al., 2019). Moreover, the drivers of seedling and sapling growth and survival vary by ontogeny, which suggests that overly coarse regeneration surveys (i.e., one size class for seedlings) may sometimes be a misleading indicator of potential forest change (Harris et al., 2024; Henry et al., 2021; Walters et al., 2020). However, we still assume that sapling composition is an indicator of future canopy tree composition. This hypothesis is likely to confounded by the wide variety of forest conditions across the eastern US including highly disturbed, multi‐aged systems versus managed even‐aged timberlands. Because we focused on advanced regeneration and potential sapling recruitment in mostly closed‐canopy forests, our analysis may overestimate what the relative abundance of shade‐tolerant species would be in the decades following a severe disturbance. Therefore, our assessment of C replacement potential may be optimistic given that shade‐tolerant species tended to be associated with greater C stocks. In future work, potential tree composition could be predicted from sapling composition using statistical techniques to estimate tree recruitment, growth and mortality (Schultz et al., 2022); or by using stand projection models such as the Forest Vegetation Simulator (Crookston & Dixon, 2005) to predict C trajectories under different natural disturbance (Murray et al., 2024) or timber harvesting scenarios.

Other sources of uncertainty are (1) that our analysis includes the C stock model and (2) the sparse nature of the RI plots, especially compared to the full pool of FIA plots (one plot per 194 km^2^ vs. 24 km^2^). It is perhaps unsurprising that the majority of plot‐level variability in live tree C stocks (58%) was not explained by our model given the heterogeneity of forests in the study area and the simplified representation of species composition (i.e., ordination scores) that we used. We emphasize that we were not attempting to develop a robust model of future C stocks, but rather to develop an indicator to assist with interpreting and managing tree regeneration patterns. The RI dataset was developed to provide more robust indicators of regeneration with its value demonstrated in regional‐scale studies (Harris et al., 2022, 2024; Vickers, McWilliams, Knapp, D'Amato, Dey, et al., 2019). The use of the RI dataset was warranted in this particular analysis to provide a more accurate indicator of sapling recruitment while enabling assessment of DWM C. Additionally, repeating our analysis using FIA's standard single seedling size class yielded broadly similar results (see Appendix S1) suggesting that extending our methodology to the full set of FIA plots that collect coarse seedling abundance measurements may be warranted in future work.

## 5 CONCLUSION

To effectively develop forest carbon management strategies, it may be important to both assess the implications of tree regeneration for future forest composition and to connect these compositional shifts to changes in ecosystem structure and function related to carbon dynamics. In this study, a sapling recruitment indicator was developed from a regeneration inventory and applied to assess a potential quantification of C resiliency. We focused our analysis on aboveground C stocks, but the impact of potential compositional shifts on other aspects of forest structure and function also deserve consideration particularly in the context of resilience to future conditions induced by climate change. This is consistent with broader calls for forest C stewardship practices that not only focus on maintaining and increasing C storage, but also long‐term C resilience in the face of changing climate and disturbance regimes (Anderegg et al., 2020; D'Amato et al., 2022; Vilsack, 2022). Our results indicate that aboveground C replacement potential is to some extent predictable by physiographic setting, current C stocks and forest type group. These associations could be used to identify areas that are most vulnerable to losing C storage capacity and prioritize these areas when developing management strategies to increase tree regeneration and associated sapling recruitment success.

## AUTHOR CONTRIBUTIONS

**Lucas B. Harris:** Conceptualization (equal); formal analysis (lead); methodology (equal); writing – original draft (lead). **Christopher W. Woodall:** Conceptualization (equal); methodology (equal); writing – review and editing (equal). **Anthony W. D'Amato:** Conceptualization (equal); methodology (equal); writing – review and editing (equal).

## CONFLICT OF INTEREST STATEMENT

The authors declare no conflict of interest.

## Supporting information


Appendix S1.

## Data Availability

This study used publicly available forest inventory data from the USDA Forest Service FIA database (https://doi.org/10.7809/b‐e.00079).
