# Supplementary material for: Sapling recruitment as an indicator of carbon resiliency in forests of the northern USA
Source: Ecol Evol. 2024 Aug 7;14(8):e70077. doi: 10.1002/ece3.70077 (PMC11304899; doi:10.1002/ece3.70077)
Supplement: Supplementary file 1 — Appendix S1. [file ECE3-14-e70077-s001.docx]

Supporting Information for:

**Sapling recruitment as an indicator of carbon resiliency in forests of the northern USA**

*Sapling recruitment models*

Following the methods of Harris *et al* (2022), Boosted Regression Tree (BRT) models (Elith et al., 2008) were developed to predict the presence or absence of recruited saplings in Regeneration Indicator (RI, McWilliams et al., 2015) subplot measurements (at Time 2, the most recent plot measurement) as a function of seedling abundance by RI height class in the previous subplot measurement (Time 1, 5–7 years prior) as well as other stand and site-level influences (see Table 1 of Harris *et al* 2022). We used a more recent version of the FIA database (v1.9.1, accessed 10/13/2023) compared with Harris et al. (2022) when building sapling recruitment models, and also applied slightly different selection criteria: all four subplots within a plot had to contain two RI measurements and be fully sampled for downed woody material (DWM) for a given subplot to be included. Subplots that had been harvested between the Time 1 and Time 2 measurements were included in the process of modeling sapling recruitment following (Harris et al. (2022), but were subsequently excluded from analyses of C replacement presented in the main text. As a result, some of the sapling recruitment models included percentage of tree basal area harvested as a predictor but this value was zero by definition for all plots in the C replacement analysis. BRT models were implemented with the “dismo” R package (Hijmans et al., 2021) using a tree complexity value of 4, a learning rate of 0.001, and 10 cross-validation folds. Variable selection was performed using the “gbm.simplify” function, which selects variables based on change in predictive deviance when variables of low importance are iteratively removed (Elith et al., 2008).

Harris *et al* (2022) had chosen individual species for analysis if sapling recruitment was present in at least 50 RI subplots in the most recent measurement cycle, but we selected a lower threshold of 30 subplots in order to include more species. Based on this 30-subplot threshold, 15 species were selected (Table S1). Because decreasing this threshold meant an increased risk of model overfitting for sparsely-represented species, we examined model accuracy and partial dependence plots (Friedman, 2001) to assess overfitting. Model accuracy as quantified using crossfold validation did not decrease for species with less abundant sapling recruitment (Table S2). In fact, species represented by 30−49 subplots with sapling recruitment produced models that had a marginally higher average AUC (0.89) than species that met the original 50-subplot threshold. In addition, visual inspection of partial dependence plots did not reveal notable differences in overfitting (i.e., as indicated by jagged lines or ecologically implausible relations) between species that met or did not meet the 50-subplot sapling recruitment threshold. These two lines of evidence suggest that relaxing the sapling recruitment threshold to 30 subplots did not lead to model overfitting that might bias the results.

As in Harris *et al* (2022), we compared two versions of each model: one using abundance within the six RI height classes, and one using standard FIA seedling tallies with a single height class. Using the RI height classes improved predictions of sapling recruitment for 14 of 15 species as quantified by the Area Under the receiver operating characteristic Curve (AUC) (Table S2). These results confirmed the findings of Harris *et al* (2022), who found that using the RI height class data improved sapling recruitment predictions. Model accuracy was similar to that of Harris *et al* (2022) for the five species included in that analysis.

The individual-species models of sapling recruitment contained 2–10 variables each, and in 14 of 15 cases the abundance of either Class 6 (>3.0 m tall) or Class 5 (1.5–3.0 m tall) seedlings was the most important variable in the model (Table S2). Total live tree basal area (BA) appeared in nine models and had a negative relationship with sapling recruitment likelihood in each case. Together with conspecific BA (two models) and total tree density (three models), the live tree BA relationships speak to the importance of the canopy openings in driving sapling recruitment. Seven models contained at least one climate variable. Other influences on sapling recruitment included litter and duff depth; grass, forb and shrub cover; and harvesting intensity (Table S2). Spearman rank correlations (*r_s_*) between predictors were generally <0.50, suggesting no major issues with multicollinearity. The only pair of predictors with *r_s_* > 0.50 were Class 4 and Class 5 seedlings of *Abies balsamea (r_s_* = 0.51)*.*

Because we wanted to predict sapling recruitment for all tree species to get a full picture of species composition, we also created a model of sapling recruitment for all species combined using the same modeling approach as for the individual species models. This “all-species” model was built from the 5,822 RI subplots meeting our analysis criteria which contained seedlings at Time 1 (Table S1). Using the RI height classes improved model AUC compared with using standard FIA seedling abundance (Table S2). The accuracy of the six-class all-species model was lower than all but one of the single-species models but not substantially so, suggesting that recruitment likelihood could be robustly estimated from seedling abundance across species using a general model (Table S2). Class 5 seedling abundance was the most influential variable in the all-species model, with Class 6 and Class 4 also included (Table S2). The all-species model also included live tree BA, elevation and mean temperature from May–October (Table S2).

After the BRT models had been built and evaluated, they were used to predict likelihood of sapling recruitment (on a 0–1 scale) for each RI subplot based on seedling abundance by RI size class and species at Time 2. Recruitment likelihood was assumed to be 0 if no seedlings of a given species were found in a subplot. These recruitment likelihoods (on a 0–1 scale) were averaged among the four subplots within each plot to obtain plot-level recruitment likelihood for each species.

Table S1. Individual species for which sapling recruitment was modeled, number of subplots (plots) in which seedlings and sapling recruitment was present, and the mean (standard deviation) of sapling recruits (stems ha^-1^) when present.

| Species | Seedlings | Sapling recruitment | Recruitment density |
| --- | --- | --- | --- |
| *Abies balsamea* | 1446 (574) | 201 (151) | 1194 (787) |
| *Acer rubrum* | 2543 (1105) | 127 (114) | 1295 (1139) |
| *Fagus grandifolia* | 866 (408) | 117 (89) | 1146 (781) |
| *Picea rubens* | 469 (232) | 74 (57) | 1121 (728) |
| *Acer saccharum* | 1344 (593) | 56 (49) | 1032 (730) |
| *Populus tremuloides* | 452 (300) | 52 (37) | 2394 (2135) |
| *Acer pensylvanicum* | 676 (321) | 44 (42) | 1145 (924) |
| *Fraxinus nigra* | 368 (187) | 40 (33) | 1111 (671) |
| *Fraxinus americana* | 1117 (539) | 38 (30) | 1209 (1476) |
| *Thuja occidentalis* | 343 (155) | 38 (30) | 975 (490) |
| *Picea mariana* | 240 (120) | 35 (27) | 847 (408) |
| *Ostrya virginiana* | 494 (274) | 34 (30) | 1177 (798) |
| *Betula papyrifera* | 340 (236) | 33 (31) | 1100 (965) |
| *Prunus serotina* | 1007 (596) | 30 (26) | 1531 (1416) |
| *Pinus strobus* | 375 (205) | 32 (24) | 1135 (922) |
| All species | 5822 (1900) | 1202 (800) | 1468 (1399) |

Table S2. Accuracy (Area Under the Curve, AUC) for sapling recruitment models with one as opposed to six seedling size classes, and variables retained with their importance for the six-class models.

| Species | AUC one | AUC six | Variables |  |
| --- | --- | --- | --- | --- |
| *Abies balsamea* | 0.78 (0.02) | 0.88 (0.01) | Class 5 (53%), total basal area (BA) (17%), total density (12%), Class 4 (10%), May–Oct. precipitation (8%) |  |
| *Acer rubrum* | 0.86 (0.01) | 0.91 (0.01) | Class 6 (50%), total BA (23%), Class 5 (14%), total density (12%) |  |
| *Fagus grandifolia* | 0.76 (0.03) | 0.87 (0.01) | Total BA (38%), Class 6 (35%), Class 5 (27%) |  |
| *Picea rubens* | 0.74 (0.03) | 0.88 (0.02) | Class 5 (54%), total BA (46%) |  |
| *Acer saccharum* | 0.79 (0.03) | 0.86 (0.02) | Class 6 (33%), BA harvested (14%), Class 5 (14%), conspecific BA (14%), total density (13%), forb cover (12%) |  |
| *Populus tremuloides* | 0.90 (0.02) | 0.89 (0.03) | Class 5 (23%), BA harvested (18%), Nov.–April precipitation (11%), total BA (10%), shrub cover (9%), Class 4 (7%), duff depth (7%), litter depth (6%), grass cover (5%), conspecific BA (4%) |  |
|  |  |  |  |  |
| *Fraxinus nigra* | 0.72 (0.04) | 0.89 (0.03) | Class 6 (67%), coarse woody material volume (33%) |  |
| *Acer pensylvanicum* | 0.76 (0.03) | 0.85 (0.02) | Class 6 (56%), total BA (44%) |  |
| *Fraxinus americana* | | 0.75 (0.03) | 0.87 (0.02) | Class 6 (43%), May–Oct. precipitation (21%), aspect (21%), May–Oct. temperature (15%) |
| *Ostrya virginiana* | 0.71 (0.04) | 0.87 (0.03) | Class 6 (53%), May–Oct. precipitation (27%), grass cover (20%) |  |
| *Betula papyrifera* | 0.73 (0.04) | 0.83 (0.03) | Class 6 (35%), total BA (33%), Nov.–April precipitation (32%) |  |
| *Thuja occidentalis* | 0.75 (0.03) | 0.88 (0.03) | Class 5 (61%), total BA (39%) |  |
| *Picea mariana* | 0.70 (0.04) | 0.92 (0.02) | Class 5 (69%), Nov.–April temperature (31%) |  |
| *Prunus serotina* | 0.82 (0.04) | 0.93 (0.02) | Class 5 (34%), BA harvested (28%), Class 6 (22%), total BA (16%) |  |
| *Pinus strobus* | 0.79 (0.03) | 0.93 (0.02) | Class 5 (44%), litter depth (19%), Nov.–April maximum vapor pressure deficit (19%), other species Class 4 (18%) |  |
| All species | 0.75 (0.01) | 0.83 (0.01) | Class 5 (49%), Class 6 (19%), total BA (15%), Class 4 (6%), elevation (5%), May–Oct. temperature (5%) |  |

*Overall species composition*

Overall, species composition differed for live tree C storage as compared with three measures of regeneration composition: sapling abundance, seedling abundance (standard FIA tallies, stems < 2.5 cm DBH and ≥ 15.2 cm or ≥ 30.5 cm tall for softwood and hardwood species, respectively) and likelihood of sapling recruitment based on RI seedling abundance (“recruitment composition”). Some species had notably high regeneration abundance as compared with C abundance, for example *Abies balsamea* and *Fagus grandifolia* (Table S3). Other species had lower regeneration abundance than C abundance, as was true for all *Quercus* species. Moreover, seedling species composition differed depending on whether it was based on standard FIA seedling tallies or modeled recruitment likelihood. Species that were more abundant according to recruitment likelihood than seedling tallies included *Quercus rubra*, *Betula papyrifera*, and *Prunus serotina*. Species that were more abundant according to standard seedling tallies included *Acer saccharum*, *Populus tremuloides* and *Fraxinus americana*. The “other species” category, made up of tree species that represented <1% of both live tree C and recruitment composition, made up 13.1% of live tree C and 10.4% of standard seedling tallies yet 18.8% of recruitment likelihood.

Table S3. Tree species composition (%) across all plots by live tree carbon (C), sapling abundance, seedling abundance and likelihood of sapling recruitment.

| Species | Tree C | Sapling | Seedling (abundance) | Seedling (recruitment) |
| --- | --- | --- | --- | --- |
| *Acer rubrum* | 10.9 | 9.3 | 7.5 | 6.5 |
| *Acer saccharum* | 8.1 | 7.0 | 5.5 | 3.4 |
| *Abies balsamea* | 5.7 | 12.8 | 11.3 | 9.5 |
| *Populus tremuloides* | 5.6 | 4.1 | 1.9 | 1.2 |
| *Quercus rubra* | 5.4 | 1.0 | 1.5 | 3.3 |
| *Picea rubens* | 3.8 | 3.7 | 3.3 | 3.5 |
| *Quercus alba* | 3.6 | 1.2 | 1.6 | 1.4 |
| *Pinus strobus* | 3.5 | 1.5 | 1.8 | 1.4 |
| *Thuja occidentalis* | 3.5 | 2.0 | 2.3 | 1.7 |
| *Picea mariana* | 3.4 | 3.5 | 3.3 | 2.8 |
| *Fagus grandifolia* | 3.3 | 8.4 | 9.5 | 8.2 |
| *Betula alleghaniensis* | 3.1 | 1.5 | 1.6 | 2.6 |
| *Tsuga canadensis* | 2.9 | 2.3 | 1.7 | 1.6 |
| *Betula papyrifera* | 2.9 | 2.3 | 1.0 | 1.3 |
| *Larix laricina* | 2.2 | 1.9 | 1.0 | 1.4 |
| *Quercus velutina* | 2.2 | 0.4 | 0.8 | 1.0 |
| *Prunus serotina* | 2.1 | 1.4 | 1.7 | 0.8 |
| *Fraxinus americana* | 1.8 | 2.3 | 6.1 | 2.1 |
| *Quercus prinus* | 1.4 | 0.2 | 0.5 | 0.5 |
| *Fraxinus nigra* | 1.3 | 2.7 | 3.4 | 2.6 |
| *Liriodendron tulipifera* | 1.3 | 0.3 | 0.1 | 0.4 |
| *Populus grandidentata* | 1.3 | 0.5 | 0.2 | 0.4 |
| *Quercus macrocarpa* | 1.3 | 0.5 | 0.3 | 0.4 |
| *Tilia americana* | 1.2 | 0.5 | 0.3 | 0.8 |
| *Betula lenta* | 0.9 | 1.6 | 1.3 | 1.7 |
| *Ulmus americana* | 0.9 | 1.6 | 1.7 | 2.4 |
| *Fraxinus pennsylvanica* | 0.7 | 0.9 | 2.0 | 2.0 |
| *Ostrya virginiana* | 0.6 | 3.8 | 2.2 | 1.8 |
| *Celtis occidentalis* | 0.5 | 1.1 | 2.1 | 1.9 |
| *Acer pensylvanicum* | 0.4 | 2.8 | 3.2 | 2.9 |
| *Carya cordiformis* | 0.4 | 0.4 | 0.7 | 1.3 |
| *Sassafras albidum* | 0.3 | 1.1 | 1.1 | 1.0 |
| *Ulmus rubra* | 0.2 | 0.6 | 0.9 | 1.3 |
| *Carpinus caroliniana* | 0.1 | 1.4 | 1.6 | 1.5 |
| *Acer spicatum* | 0.0 | 0.4 | 2.0 | 2.3 |
| *Prunus virginiana* | 0.0 | 0.3 | 2.7 | 2.7 |
| other | 13.1 | 12.7 | 10.4 | 18.8 |


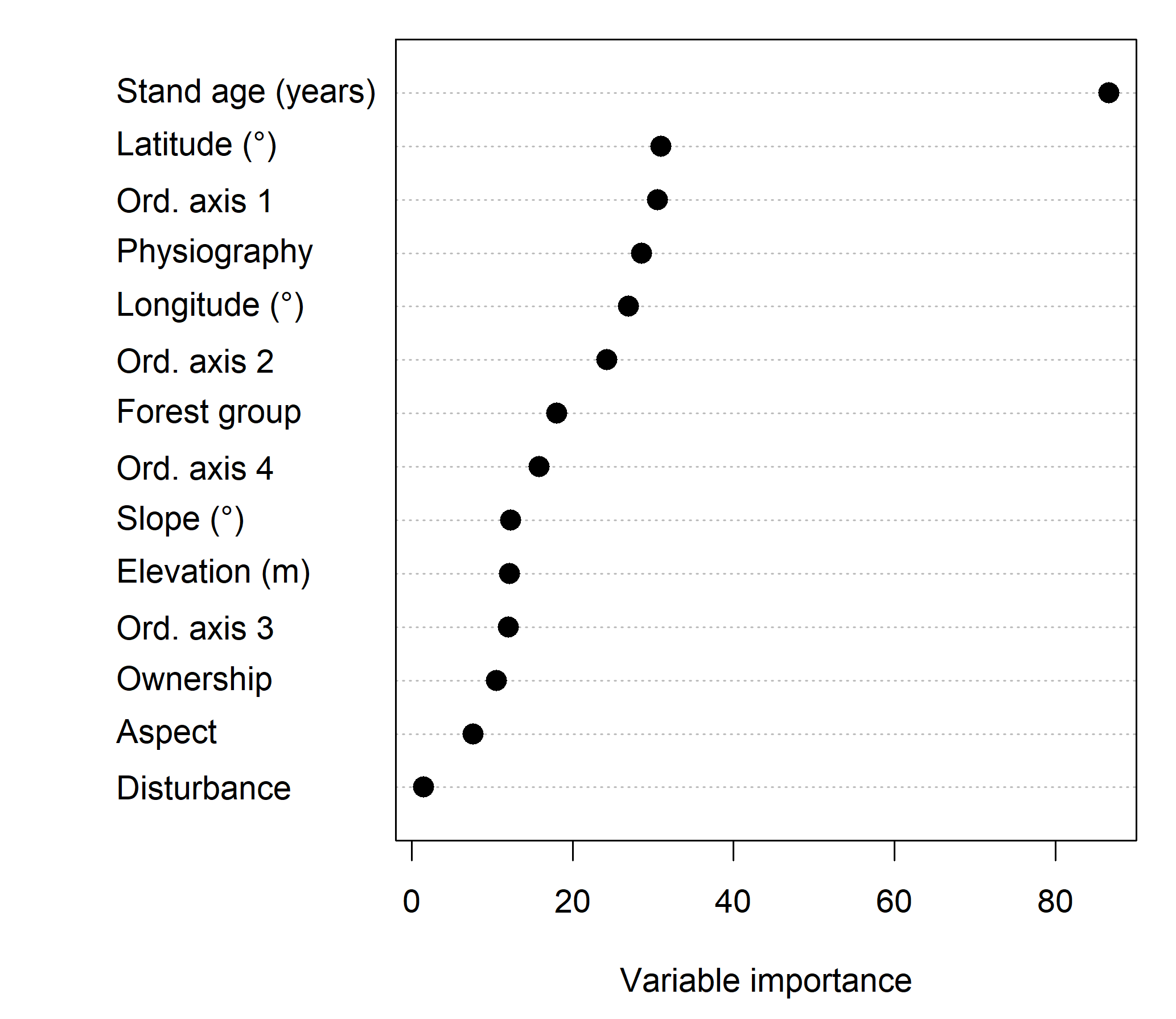


Fig. S1. Variable importance from the statistical model of live aboveground tree (C) stocks. “Ord. axis” variables are values from each of the four ordination axes that represent species composition.


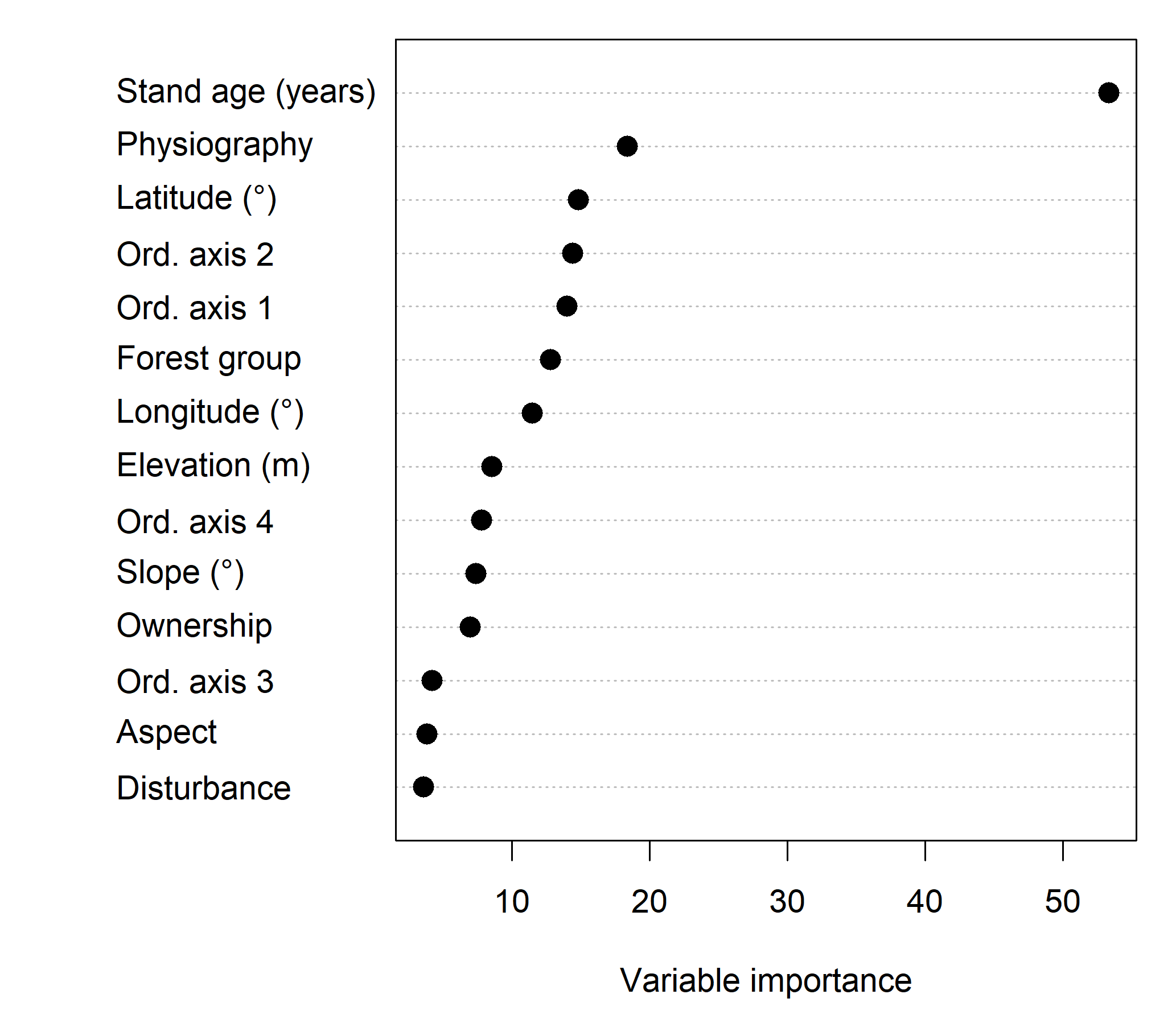


Fig. S2. Variable importance from the statistical model of total aboveground tree carbon (C) storage including live trees, snags, and downed woody material. “Ord. axis” variables are values from each of the four ordination axes that represent species composition.


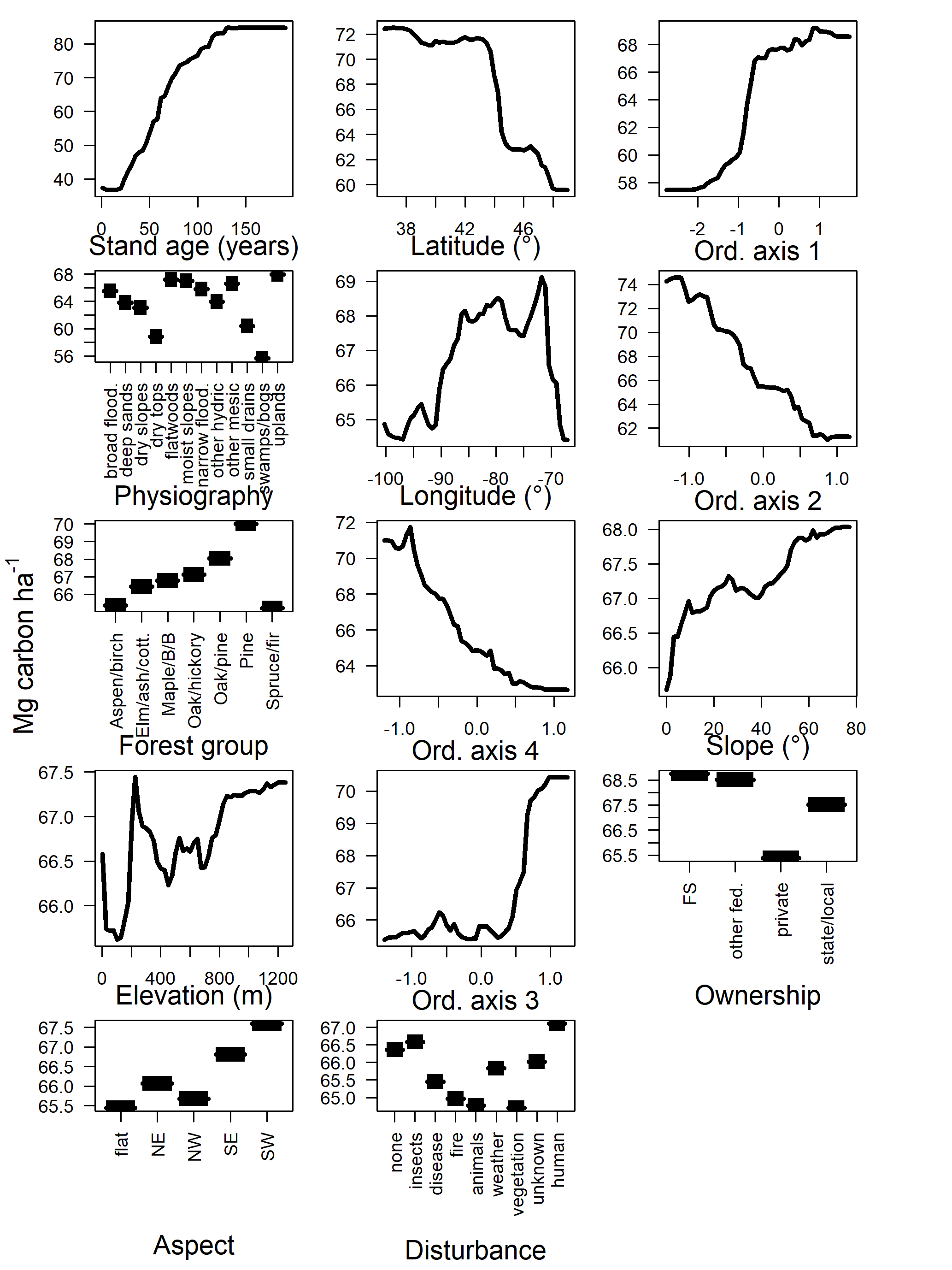


Fig. S3. Partial dependence plots from the statistical model of live tree carbon (C) storage, showing the marginal effect of each individual variable in the model in order of importance (most important variable at top left). Abbreviations are as follows: ord. = ordination, flood. = floodplain, cott. = cottonwood, Maple/B/B = Maple/Beech/Birch, FS = Forest Service, fed. = federal.


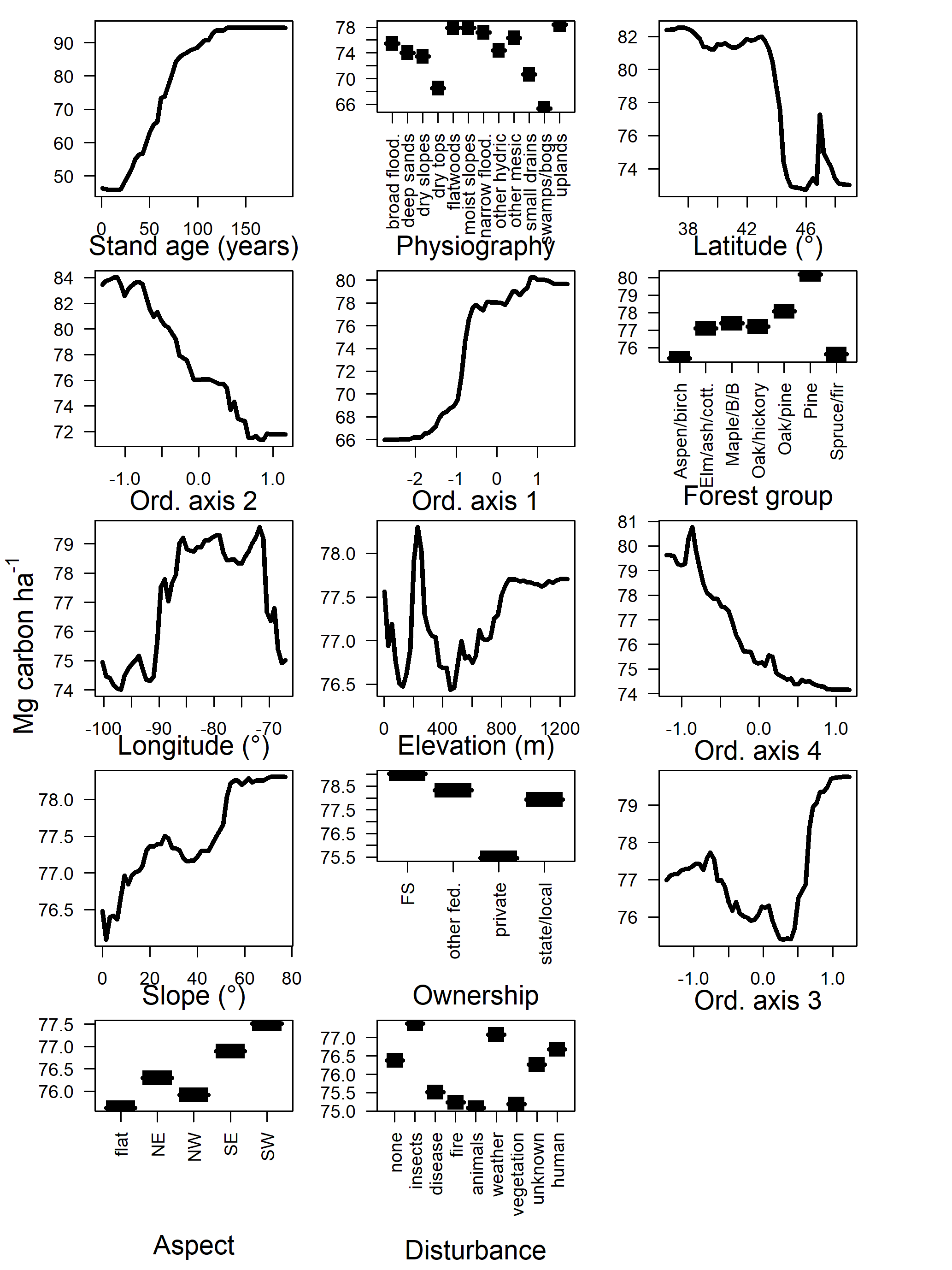


Fig. S4. Partial dependence plots from the statistical model of total carbon (C) storage, showing the marginal effect of each individual variable in the model in order of importance (most important variable at top left). Abbreviations are as follows: ord. = ordination, flood. = floodplain, cott. = cottonwood, Maple/B/B = Maple/Beech/Birch, FS = Forest Service, fed. = federal.

*Effects of stand age and categorizing C stock differences*

In the analyses of variability in and effects on C replacement presented in Fig. 4 and 5, we made two decisions: (1) analyzing difference in C stocks at a stand age of 100 years and (2) categorizing C stock differences into “loss”, “replacement” and “gain” using arbitrary thresholds of −5 and +5 Mg C ha^1^.

To assess whether results were robust to the use of stand ages other than 100 years, we performed the same analysis at stand ages of 20, 40, 60, 80, 100, 120 and 140 years. All of the variables that were significant (p < 0.05) at 100 years were also significant across 60–140-year stand ages and four of seven were significant across the full range of 20–140 years (Table S4). Therefore, results shown in the main text apply to a range of maturing to mature stand ages of 60–140 years, with some differences observed at younger predicted stand ages of 20 and 40 years.

We assessed the impact of categorizing C differences into C replacement categories by testing whether the same variables that varied significantly by C replacement class also varied significantly by difference in predicted C stocks (a numeric variable, in Mg ha^-1^). Significance was assessed using Spearman rank correlations for numeric variables and Kruskal-Wallis tests for categorical variables, with a Holm-Bonferroni correction applied for multiple comparisons. As shown by comparing Tables S4 and S5, results were highly similar when considering difference in C stocks rather than C replacement categories. Importantly, all of the variables presented as significant in Fig. 5 were also significant when tested using difference in C stocks. Relationships between these variables and difference in C stocks (Fig. S5) were similar to those presented in Fig. 5, albeit more difficult to visualize in some cases. C in downed woody material was the one variable that differed significantly according to difference in C but not C replacement categories (Figure S5h), showing a weakly negative relationship with difference in C.

Table S4. P-values indicating whether carbon (C) replacement categories (gain, replacement and loss) varied significantly by stand and site-level factors across different predicted stand ages.

| Variable | 20 yr. | 40 yr. | 60 yr. | 80 yr. | 100 yr. | 120 yr. | 140 yr. |
| --- | --- | --- | --- | --- | --- | --- | --- |
| Latitude | 0.001 | 0.018 | <0.001 | <0.001 | <0.001 | 0.011 | 0.011 |
| Longitude | 0.058 | 1 | 0.553 | 1 | 1 | 1 | 1 |
| Elevation | 0.355 | 1 | 0.105 | 0.078 | 0.369 | 0.514 | 0.947 |
| Stand age | <0.001 | <0.001 | <0.001 | <0.001 | <0.001 | <0.001 | <0.001 |
| Slope | 0.005 | 0.044 | <0.001 | <0.001 | <0.001 | <0.001 | <0.001 |
| Live tree C | <0.001 | <0.001 | <0.001 | <0.001 | <0.001 | <0.001 | <0.001 |
| Standing dead C | 0.037 | 0.013 | 0.021 | 1 | 1 | 1 | 1 |
| Downed woody material C | 0.001 | <0.001 | <0.001 | 0.019 | 0.297 | 0.064 | 0.045 |
| Mean temperature | 1 | 1 | 1 | 1 | 1 | 1 | 1 |
| Total precipitation | 1 | 1 | 1 | 1 | 1 | 1 | 1 |
| Maximum vapor pressure deficit | 1 | 1 | 1 | 1 | 1 | 1 | 1 |
| Δ live tree C | 1 | 1 | 1 | 1 | 1 | 1 | 1 |
| Aspect | 0.355 | 0.408 | 0.001 | 0.001 | 0.001 | 0.001 | 0.001 |
| Physiography | 0.262 | 0.098 | 0.001 | 0.001 | 0.001 | 0.001 | 0.001 |
| Disturbance type | 0.355 | 0.408 | 0.539 | 1 | 1 | 1 | 1 |
| Forest group | 0.022 | 0.408 | 0.001 | 0.001 | 0.001 | 0.001 | 0.001 |

Table S5. P-values indicating whether difference in predicted carbon (C) based on recruitment vs. tree composition varied significantly by stand and site-level factors across different predicted stand ages.

| Variable | 20 yr. | 40 yr. | 60 yr. | 80 yr. | 100 yr. | 120 yr. | 140 yr. |
| --- | --- | --- | --- | --- | --- | --- | --- |
| Latitude | 0.136 | 1 | <0.001 | <0.001 | <0.001 | <0.001 | 0.001 |
| Longitude | 0.001 | 0.146 | 0.069 | 1 | 1 | 1 | 1 |
| Elevation | 1 | 1 | 0.156 | 1 | 1 | 1 | 1 |
| Stand age | <0.001 | <0.001 | <0.001 | <0.001 | <0.001 | <0.001 | <0.001 |
| Slope | <0.001 | <0.001 | <0.001 | <0.001 | <0.001 | <0.001 | <0.001 |
| Live tree C | <0.001 | <0.001 | <0.001 | <0.001 | <0.001 | <0.001 | <0.001 |
| Standing dead C | <0.001 | <0.001 | 0.001 | 1 | 1 | 1 | 0.976 |
| Downed woody material C | <0.001 | <0.001 | <0.001 | 0.005 | 0.023 | 0.009 | 0.011 |
| Mean temperature | 1 | 1 | 1 | 1 | 1 | 1 | 1 |
| Total precipitation | 1 | 1 | 1 | 1 | 1 | 1 | 1 |
| Maximum vapor pressure deficit | 1 | 1 | 1 | 1 | 1 | 1 | 1 |
| Δ live tree C | 1 | 1 | 1 | 1 | 1 | 1 | 1 |
| Aspect | 0.022 | 0.057 | <0.001 | <0.001 | <0.001 | <0.001 | <0.001 |
| Physiography | 0.373 | 1 | <0.001 | <0.001 | <0.001 | <0.001 | <0.001 |
| Disturbance type | 1 | 1 | 0.115 | 1 | 1 | 0.625 | 0.237 |
| Forest group | 0.373 | 0.304 | 0.001 | <0.001 | <0.001 | <0.001 | <0.001 |


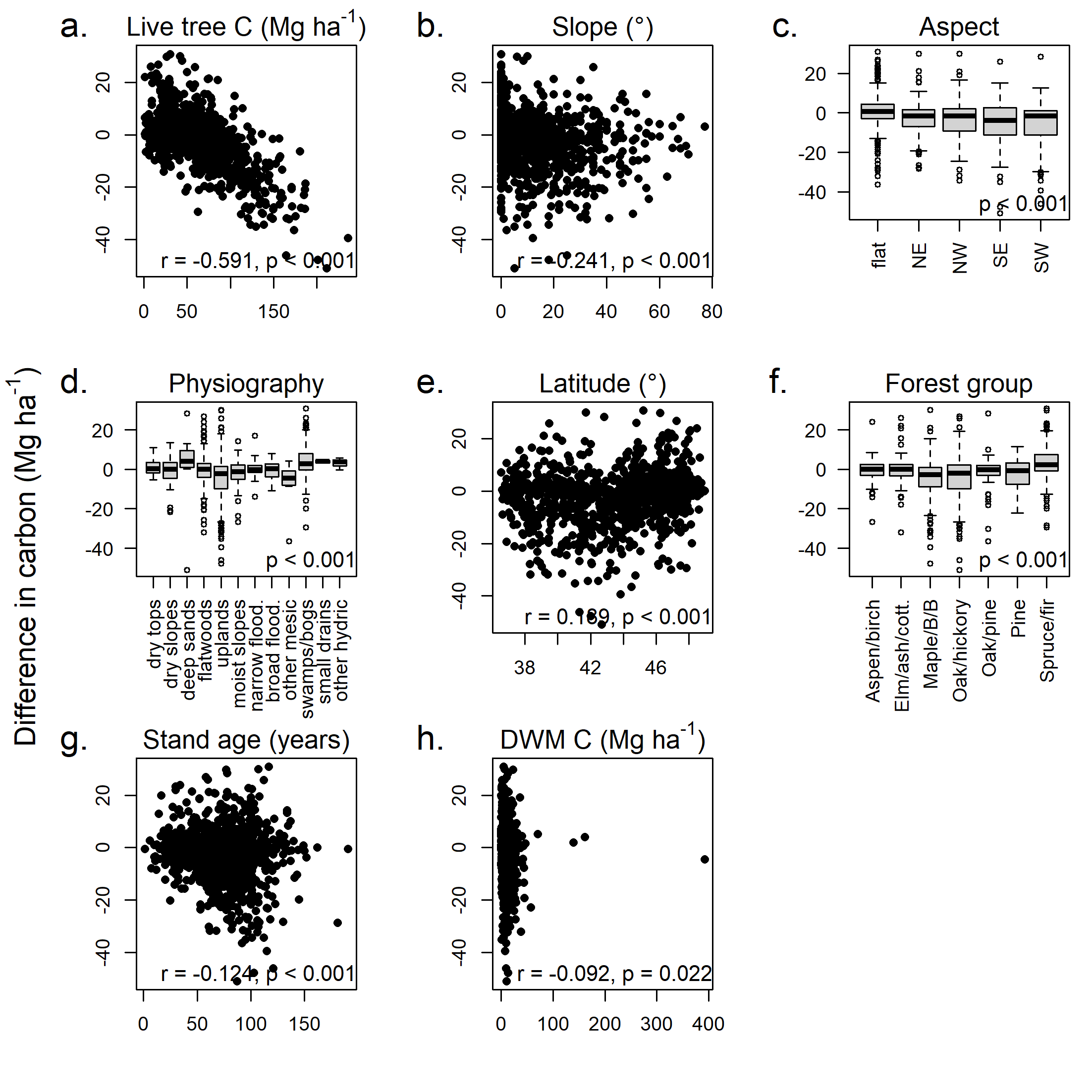


Fig. S5. Statistically significant (p < 0.05) relationships between individual variables and difference in predicted C stocks. Compare with Fig. 5 in the main text, which shows significant relationships when difference in C stocks is categorized. P-values are shown in all panels, and Spearman rank correlation coefficients are shown for numeric variables.

*Effects of using one size class for seedlings*

We chose to use the RI dataset for analyzing sapling recruitment as opposed to the broader pool of FIA plots because the six seedling size classes available in the RI dataset have been shown to be valuable for assessing the robustness of regeneration (Harris et al., 2024, 2022; Vickers et al., 2019) and because the RI measurements coincide with detailed vegetation and downed dead wood (DDW) surveys not otherwise conducted by FIA. However, the much lower plot density of the RI plots compared with all FIA plots (one plot per 194 km^2^ vs. 24 km^2^) limits the scope of inference from the RI dataset and begs the question of whether the broader pool of FIA plots could be used to analyze sapling recruitment potential and C replacement.

To identify the impact of using FIA’s standard single size class for seedlings as opposed to the six size classes available in the RI, we repeated our analysis (i.e., ordination, models of C stocks and assessments of C replacement) using the single size class sapling recruitment models instead of the six-class models. The resulting model of live tree C stocks had a pseudo-*r^2^* of 42.0%, comparable to the 41.8% using the RI seedling classes, and effects of tree species composition on C stocks were similar (compare Fig. S6 with Fig. 4). Compositional shifts between trees and sapling recruitment potential showed only minor differences using one vs. six seedling height classes for some forest groups (aspen/birch, elm/ash/cottonwood, oak/pine and spruce/fir) whereas moderate differences were evident for other groups (maple/beech/birch, oak/hickory and pine) (compare Fig. S6 with Fig. 4). Associations with C replacement shown in Fig. 6 were similar when using one size class for seedlings (Fig. S7) and the same variables were significant across the two analyses with the only difference being that longitude differed significantly when using one size class (Fig S7h).


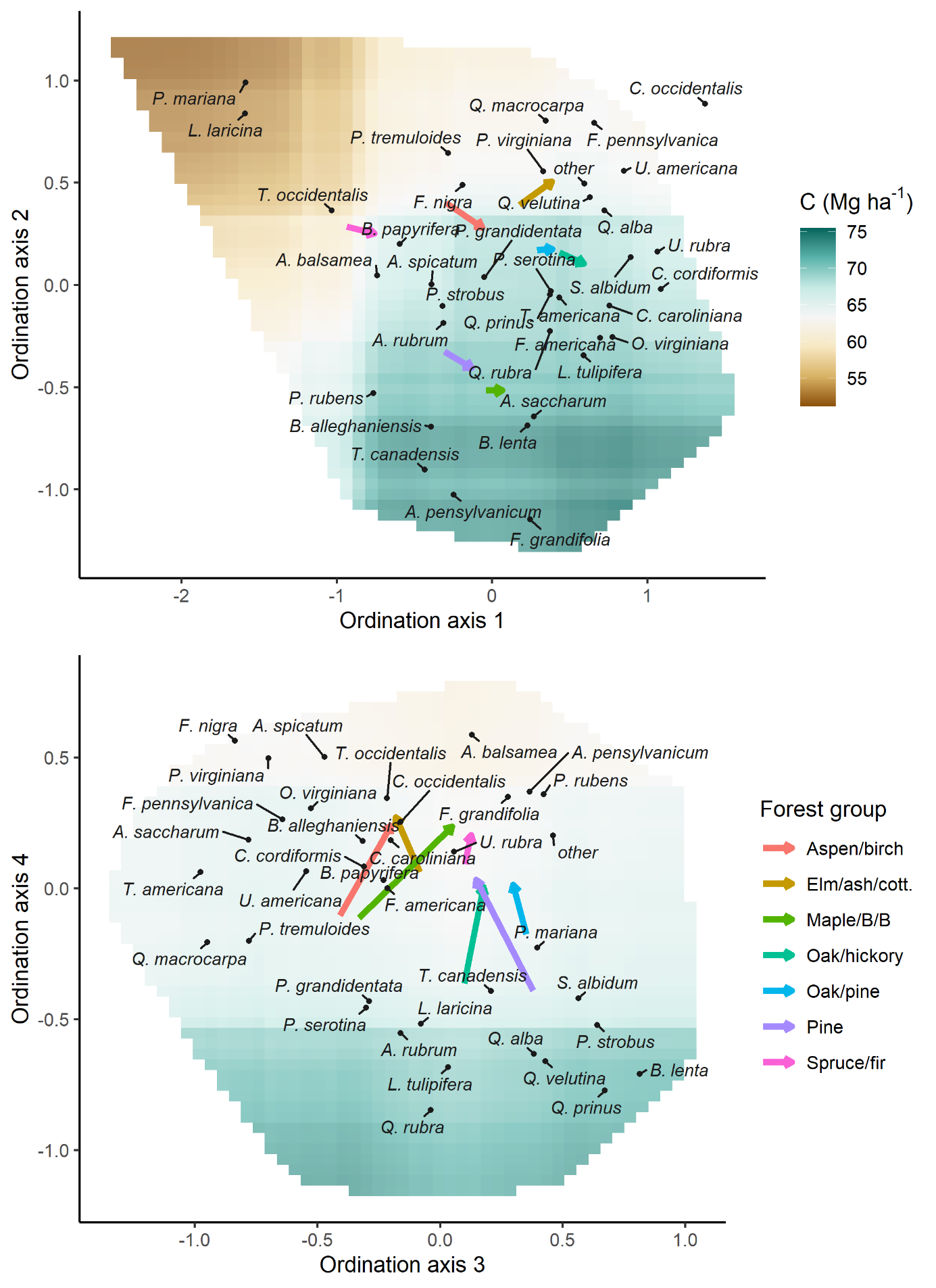


Fig. S6. Results of an ordination performed using predicted sapling recruitment from standard Forest Inventory and Analysis seedling abundance in one size class as opposed to the six seedling size classes tallied in Regeneration Indicator plots. Compare with Fig. 4 in the main text, which uses six seedling size classes tallied in Regeneration Indicator plots. Ordination scores are shown for tree species, with partial dependence plots displaying the marginal influence of species composition on predicted live aboveground tree carbon (C) stocks. Arrows show average species composition of live tree C (beginning of arrow) and recruitment composition (end of arrow) by forest group. Longer arrows represent forest groups where the composition of live tree C varied more from the composition of seedlings.


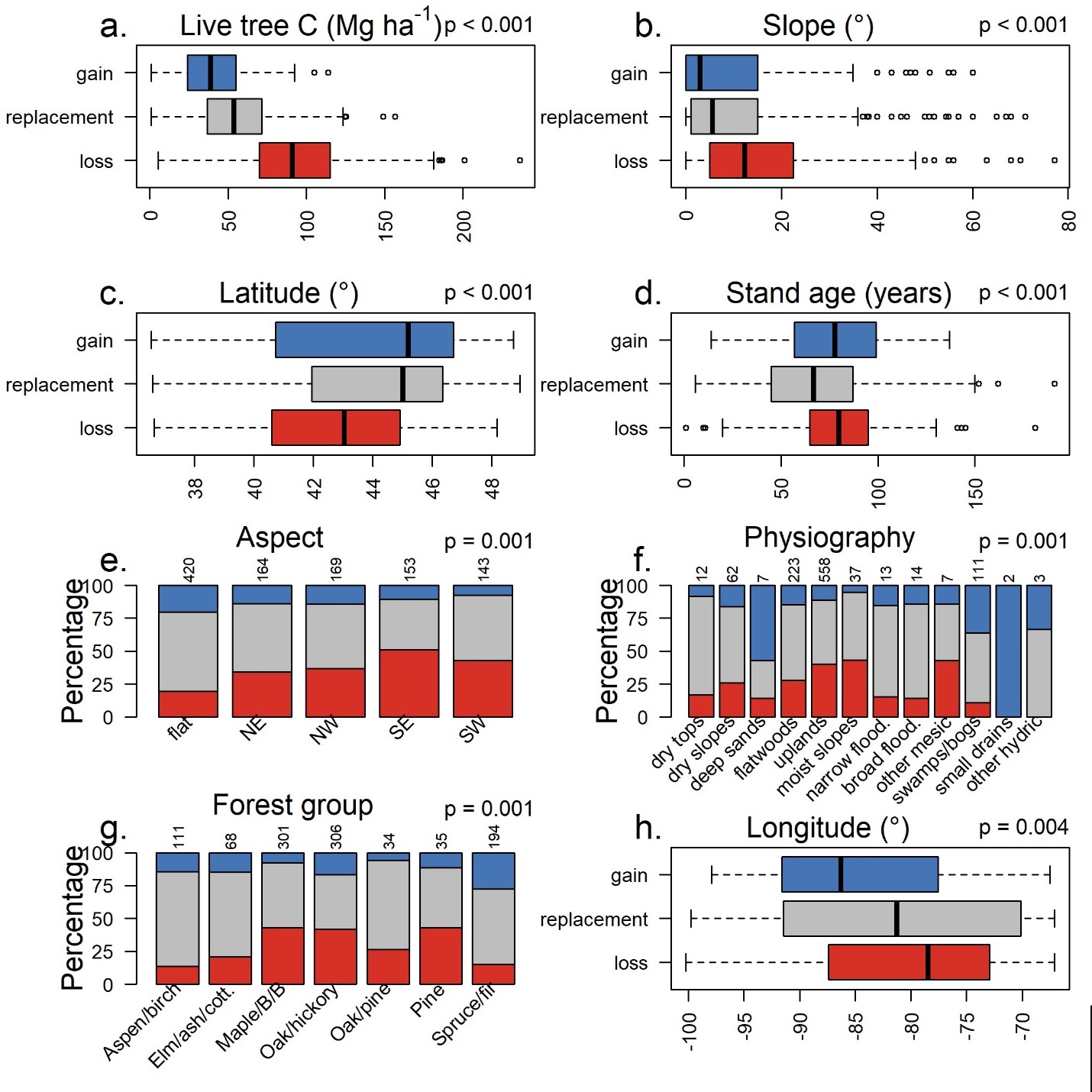


Fig. S7. Variation in carbon (C) replacement, i.e., plots predicted to lose, replace or gain C stocks based on recruitment composition calculated from models with one seedling size class compared to tree composition at a predicted stand age of 100 years, by physiography, geography and stand attributes. Compare with Fig. 6 which shows the same analysis but with sapling recruitment modeled using the six seedling size classes in the Regeneration Indicator dataset. Variables are ordered alphabetically by significance (Kruskal-Wallis test for numeric variables and Chi-squared test for categorical variables) and p-values (Holm-Bonferroni correction applied) are reported in each panel. For categorical variables, number of plots in each category is shown above each bar. Flood. = floodplain, Elm/ash/cott. = Elm/ash/cottonwood, and Maple/B/B = Maple/Beech/Birch.

**References**

Elith, J., Leathwick, J.R., Hastie, T., 2008. A working guide to boosted regression trees. The Journal of Animal Ecology 77, 802–13. https://doi.org/10.1111/j.1365-2656.2008.01390.x

Friedman, J.H., 2001. Greedy function approximation: A gradient boosting machine. The Annals of Statistics 29, 1189–1232.

Harris, L.B., Woodall, C.W., D’Amato, A.W., 2024. Relationships between juvenile tree survival and tree density, shrub cover and temperature vary by size class based on ratios of abundance. Canadian Journal of Forest Research 54, 122–133. https://doi.org/10.1139/cjfr-2023-0097

Harris, L.B., Woodall, C.W., D’Amato, A.W., 2022. Increasing the utility of tree regeneration inventories: Linking seedling abundance to sapling recruitment. Ecological Indicators 145, 109654. https://doi.org/10.1016/j.ecolind.2022.109654

Hijmans, R.J., Phillips, S., R. Leathwick, J., Elith, J., 2021. dismo: Species Distribution Modeling. https://doi.org/https://cran.r-project.org/web/packages/dismo/index.html

McWilliams, W.H., Westfall, J.A., Brose, P.H., Dey, D.C., Hatfield, M., Johnson, K., Laustsen, K.M., Lehman, S.L., Morin, R.S., Nelson, M.D., Ristau, T.E., Royo, A.A., Stout, S.L., Willard, T., Woodall, C.W., 2015. A regeneration indicator for forest inventory and analysis: history, sampling, estimation, analytics, and potential use in the Midwest and Northeast United States. U.S. Department of Agriculture, Forest Service, General Technical report NRS-148 1–74.

Vickers, L.A., McWilliams, W.H., Knapp, B.O., D’Amato, A.W., Dey, D.C., Dickinson, Y.L., Kabrick, J.M., Kenefic, L.S., Kern, C.C., Larsen, D.R., Royo, A.A., Saunders, M.R., Shifley, S.R., Westfall, J.A., 2019. Are current seedling demographics poised to regenerate northern US forests? Journal of Forestry 117, 592–612. https://doi.org/10.1093/jofore/fvz046
